# Comparative Genomic Hybridization and Transcriptome Sequencing Reveal Genes with Gain in Acute Lymphoblastic Leukemia: *JUP* Expression Emerges as a Survival-Related Gene

**DOI:** 10.3390/diagnostics12112788

**Published:** 2022-11-14

**Authors:** Jessica Alejandra Zapata-García, Alma Rocío Riveros-Magaña, Pablo Cesar Ortiz-Lazareno, Georgina Hernández-Flores, Luis Felipe Jave-Suárez, Adriana Aguilar-Lemarroy

**Affiliations:** 1 Programa de Doctorado en Ciencias Biomédicas, Centro Universitario de Ciencias de la Salud, Universidad de Guadalajara, Guadalajara C.P. 44340, Mexico; 2 División de Inmunología, Centro de Investigación Biomédica de Occidente (CIBO), Instituto Mexicano del Seguro Social (IMSS), Guadalajara C.P. 44340, Mexico; 3 Centro Universitario del Sur, Universidad de Guadalajara, Ciudad Guzmán C.P. 49000, Mexico; 4 Hospital General Zona 9, Ciudad Guzmán C.P. 49000, Mexico

**Keywords:** CGH, leukemia, ALL, biomarker, gene expression, overall survival, RNAseq

## Abstract

Acute lymphoblastic leukemia (ALL) in children or adults is characterized by structural and numeric aberrations in chromosomes; these anomalies strongly correlate with prognosis and clinical outcome. Therefore, this work aimed to identify the genes present in chromosomal gain regions found more frequently in patients with acute lymphoblastic leukemia (ALL) and ALL-derived cell lines using comparative genomic hybridization (CGH). In addition, validation of the genes found in these regions was performed utilizing RNAseq from JURKAT, CEM, and SUP-B15 cell lines, as well as expression microarrays derived from a MILE study. Chromosomes with common gain zones that were maintained in six or more samples were 14, 17, and 22, in which a total of 22 genes were identified. From them, *NT5C3B*, *CNP*, *ACLY*, and *GNB1L* maintained overexpression at the mRNA level in the cell lines and in patients with ALL. It is noteworthy that *SALL2* showed very high expression in T-ALL, while *JUP* was highly expressed in B-ALL lineages. Interestingly, the latter correlated with worse survival in patients. This provided evidence that the measurement of these genes has high potential for clinical utility; however, their expressions should first be evaluated with a sensitive test in a more significant number of patients.

## 1. Introduction

Acute lymphoblastic leukemia (ALL) results from the malignant transformation and proliferation of hematopoietic stem cells (HSCs) in the bone marrow, blood, and extramedullary sites, and it is characterized by genetic mutations [1]. It was recently reported that leukemia could also be initiated by leukemic stem cells (LSCs), which, like normal HSCs, have a cellular reservoir that drives relapse by restarting the disease after remission [2].

Although there may be predisposing factors to develop the disease, such as the presence of some syndromes (Down syndrome [3], Fanconi anemia [4], Bloom syndrome [5], ataxia telangiectasia [6]), or exposure to ionizing radiation [7]) or viruses (Epstein–Barr [8], cytomegalovirus [9], or human T-cell lymphotropic virus [10]), most people develop the disease de novo.

While ALL occurs more frequently in children, a high percentage recover; however, it represents a devastating disease when it occurs in adults [11]. Its global incidence and mortality are 5.4/100,000 and 3.3/100,000 inhabitants, respectively [12]. In Mexico, it represents a serious health problem, since the mortality rate calculated per 100,000 inhabitants is 2.1 in the pediatric population and 5.5 in adults [13].

Although the causes of the disease are not entirely clear, currently, some genes affected by genetic aberrations have become useful in clinical settings due to their valuable contribution as diagnostic and prognostic markers or for their help in monitoring minimal residual disease, among other applications [14].

Some typical anomalies useful in a clinical setting for B-ALL, in addition to hyperdiploidy and hypodiploidy, are the translocations t(9;22)(q34;q11.2) (*BCR-ABL1*) [15], t(12;21) (p13;q22) (*ETV6-RUNX1*) [16], t(1;19) (q23;p13.3) (*TCF3-PBX1*) [17], and t(5;14) (q31;q32) (*IGH-IL3*) [17]; the rearrangement t(v;11q23) (*MLL*); the intrachromosomal amplification of chromosome 21 (iAMP21) [18]; and the deletion of *CDKN2A/B* [19].

Although T-ALL shares abnormalities with B-ALL, such as the rearrangement of t(v;11q23) (*MLL*) or the deletion of *CDKN2A/B* [20], there are specific gene deregulations in this subtype, such as (1p32) (*TAL1*), (11p13) (*LMO2*), (10q24) (*TLX1/HOX11*), (5q35) (*TLX3/HOX11L2*) [21], fusion (9q34) (*NUP214-ABL1*) [22], mutations of (9q34.3) (*NOTCH1*) [23], and loss of (4q31.3) (*FBXW7*) and (10q23) (*PTEN*) [24].

In this sense, there is a growing interest and urgency to investigate changes in the genome that may be of clinical utility. Karyotyping is one of the routinely used techniques to identify these anomalies, but it has some limitations, such as the fact that it cannot detect genetic changes of less than 5 to 10 Mb and that it takes between 4 and 10 days to culture cells, visualize chromosomes, and carry out analysis, in addition to the fact that a good result depends on the quality of the chromosome preparation and the skill and experience of the cytogeneticist [25,26].

Currently, there are new molecular biology strategies that allow the determination of genomic alterations with greater sensitivity, such as molecular karyotyping, known as comparative genomic hybridization (CGH), which is characterized by allowing, with a high resolution, the identification of small numerical aberrations in a genome [27]. CGH can detect chromosomal gain or loss imbalances with much higher resolution than conventional karyotyping [28]. In addition, RNA sequencing (RNAseq) has also become one of the best strategies for analyzing the transcriptomes of individuals because it is sensitive and specific with a more profound resolution, and it provides less background noise, as well as a dynamic range of gene expression [29].

Therefore, the objective of this study is to identify the common chromosomal gain regions most frequently found in bone-marrow-derived samples from patients with ALL and the leukemia-derived cell lines JURKAT and CEM using CGH arrays; to validate expressions at the transcriptome level of the genes included in the chromosomal gains using RNAseq and microarray expression analysis; and to correlate the gene expressions with overall survival.

## 2. Materials and Methods

### 2.1. Sample Collection

The present study used bone marrow samples from ALL patients without prior treatment that were isolated by density gradient centrifugation with Ficoll-Paque™ PLUS (GE Healthcare, Chicago, IL, USA) and cryopreserved in liquid nitrogen from a previous study by the working group; the age, gender, leukemia classification, immune phenotype, and blast percentage of each patient included in this study are visualized in Table 1 of the research published by Zavala et al. [30]. The collection of the samples and project execution were approved by the IMSS National Scientific Research Commission under registration numbers R-2012-785-056, R-2019-1305-039, and R-2020-785-015.

### 2.2. Cell Line Culturing

Cell lines derived from T-ALL (JURKAT and CEM) and B-ALL (SUP-B15) were cultured in 25 cm^2^ culture flasks (Corning, Cat No. TM 3815) in RPMI 1640 (Cat.11875-093) supplemented with 10% inactivated fetal bovine serum (FBS) and 100 U/mL penicillin/1 mg/mL streptomycin (all products purchased from Life Technologies Corporation, Thermo Fisher Scientific, Waltham, MA, USA) at 37 °C in a 5% CO_2_ atmosphere. The cell lines used in this study are commercially available at the ATCC and were kindly donated by Prof. Dr. Henning Walczak (DKFZ-Heidelberg, Germany). Authentication was performed by Multiplexion GmbH (https://www.multiplexion.de accessed on 17 October 2022).

### 2.3. DNA Extraction and Array CGH

Genomic DNA was obtained using a Quick-gDNA™ MiniPrep kit (Zymo Research, Cat. D3006, CA, USA) according to the manufacturer’s instructions. DNA was stored at 4 ºC until use. Sample preparation and hybridization were performed as described in detail in the NimbleGen Arrays User Guide (Roche Applied Science, Penzberg, Germany). Briefly, 1 µg DNA from each leukemia-derived sample was taken and labeled with Cy3 (cyanine 3), and the reference DNA provided by the kit was labeled with Cy5 (cyanine 5). Subsequently, DNA probes were hybridized on a NimbleGen HG18 WG CGH Array (3 × 720 K microarrays v2.0; Roche Applied Science). After 72 h of hybridization, slides were washed and spin-dried in a SlideWasher^TM^12 (CapitalBio Corporation, Beijing, China) machine, and scanning was executed using an MS 200 NimbleGen Microarray Scanner with a resolution of 2 µm (Roche Applied Science).

The values of the Log2 ratios of the probes (Cy3/Cy5), signal intensities, and chromosomal gains and losses were calculated and visualized using DEVA software, version 1.2.1 (Roche Applied Sciences).

In brief, local polynomial regression fitting (LOESS) spatial normalization was performed, followed by Qspline normalization of the intensities of all microarrays. After ratio calculation of the test and reference samples, all data from all containers were merged into a single container. Next, a segment tree was built using SegMNT, with 500 as a maximum number of segments, a minimum number of differences between segments of 0.2 (Log2), a minimum number of probes in segments of 5, a permutation number of 10, and stringency for the percentile of 0.9.

Finally, the genes present in the gain regions were identified utilizing the UCSC Genome Browser database (https://genome.ucsc.edu/ accessed on 1 September 2022) with the GRch/38hg 38 genome version as reference. The raw and processed data obtained in this study were already deposited in the Gene Expression Omnibus (GEO) NCBI database repository under accession number GSE185274. To increase the certainty of our findings, we included samples derived from the bone marrow of adult patients with ALL without prior treatment (available under accession number GSE75671) [31]. The samples of the patients were randomly downloaded, and their corresponding IDs were as follows: GSM 1963398-1963506 (T-ALL) and GSM 1963449-1963457 (B-ALL). A detailed description of the microarray analysis can be found in the study of Castro et al. [32].

### 2.4. Circos Plot Representation of Genomic Data

The circular multitrack plots shown in were generated using genome-wide DNA copy numbers normalized to Log2 utilizing R, version 4.1.3, and RStudio software (2021.09.0). The libraries utilized were Circlize (https://cran.r-project.org/web/packages/circlize/index.html accessed on 17 October 2022), RColorBrewer (https://cran.r-project.org/web/packages/RColorBrewer/index.html accessed on 17 October 2022), GenomicRanges (https://bioconductor.org/packages/release/bioc/html/GenomicRanges.html accessed on 17 October 2022), data.table (https://cran.r-project.org/web/packages/data.table/index.html accessed on 17 October 2022), RLumShiny (https://cran.r-project.org/web/packages/RLumShiny/index.html accessed on 17 October 2022), and grDevices (https://uribo.github.io/rpkgshowcase/graphics/grDevices.html accessed on 17 October 2022). To discriminate between a region with or without gain, we use a cutoff point of 1.5.

### 2.5. RNA Dataset Analysis

RNA sequencing of JURKAT and CEM cell lines was performed using the NovaSeq 6000 Illumina platform (service from Novogene Bioinformatics Technology Co., Ltd., in Beijing, China). These sequences were deposited in the GEO NCBI repository (https://www.ncbi.nlm.nih.gov/gds with public access on 31 October 2021) and identified with accession number GSE189641 [33]. To contrast with our data, additional datasets were downloaded from JURKAT (SRP370930) [34], CEM (SRP319983) [35], and SUP-B15 (SRP319983 and SRP189893) [36]. Finally, peripheral blood derived from clinically healthy subjects (SRP281919) [37] and nonleukemia adult bone marrow data (SRP114952) [38] were also included as controls. It is important to mention that, since JURKAT and CEM were derived from peripheral blood, each cell line was compared with peripherical blood controls of similar ages. Regarding SUP-B15, this cell line was derived from the bone marrow of an 8-year-old child, so the controls used were nonleukemia bone marrow samples.

A bioinformatic analysis was carried out as follows: raw reads were analyzed using the Galaxy Europa open-source platform (usegalaxy.eu) and RStudio software (2021.09.0) utilizing Rsubread library (https://bioconductor.org/packages/Rsubread/ accessed on 18 June 2022). First, the FastQC tool (version 0.73 with galaxy0) was used to determine the quality of the sequences [39]. Subsequently, the Trimmomatic tool (version 0.38.1) [40] was used to remove ambiguous nucleotides. Clean reads were then aligned using Rsubread and human genome version hg38 (vs. 38) to obtain BAM files that were then used to count reads with the featureCounts tool (version 2.0.1 with galaxy2) [41].

Gene expression analysis was performed with DESeq2 (version 2.11.40.7 with galaxy1) [42] using FPKM (fragments per million kilobases) for normalization. The heatmap2 tool (Version 3.0.1) [43] was used to build HeatMaps using Log10 (value + 1) data transformation and the Euclidean distance method. Genes depicted in the HeatMaps had a fold change greater than 1, with statistical significance at an adjusted *p*-value ≤ 0.05.

### 2.6. Evaluation of JUP Expression by Quantitative PCR

Total RNA was isolated from peripherical mononuclear cells derived from individuals without leukemia and from JURKAT, CEM, and SUP-B15 cell lines with a Quick-RNA mini prep plus kit (Cat. No. R1058, Zymo Research, Irvine, CA, USA). Afterward, cDNA was obtained with a Transcriptor First Strand cDNA Synthesis Kit (Cat. No. 04379012001, Roche Diagnostics, Basel, Switzerland). qPCR assays were performed with a LightCycler 2.0 (Roche Diagnostics) instrument using a LightCycler FastStart DNA Master plus SYBR Green I kit (Cat. No. 03515869001, Roche Diagnostics). The sequences of the primers used to amplify *JUP* and the reference genes (*RPLP0* and *RPS18*) are represented in Table 1.

### 2.7. Expressions of Genes Included in Gain Regions in Patients from the MILE Project

To analyze the expression of each gene in patients with ALL, we used the open-access NCBI GEO database with access number GSE13159 [44,45] and R2 Genomics Analysis and Visualization Platform (http://r2.amc.nl accessed on 1 September 2022). This database is derived from a microarray design (Affymetrix HG-133 Plus 2.0) that includes samples from adults with ALL without treatment. Expressions of the 22 genes identified in the common gain regions were evaluated by comparing controls (*n* = 71) with B-ALL (*n* = 427) or T-ALL (*n* = 165) samples. A one-way ANOVA was applied, and the expressions were transformed into Log2.

### 2.8. Tree Plots of Expressions of Genes with Gains in Normal Hematopoiesis versus Leukemia Lineages

Hierarchical trees for each gene were constructed using BloodSpot, an online database of gene expression profiles and transcriptional programs for healthy and malignant hematopoiesis [46] available at www.bloodspot.eu accessed on 17 October 2022. The files used for the analysis were GSE13159 for the leukemia lineages (MILE project) [44] and GSE24759 for normal human hematopoiesis (DMAP project) [47].

### 2.9. Survival Analysis

The association between overall survival and gene expression was calculated using the Kaplan Scan tool, available in Statistical Software Environment R, version 2.4.1 (http://www.r-project.org accessed on 17 October 2022), by utilizing the GEO NCBI database with access number GSE34861 [48], which corresponds to adult B-ALL samples. Data with *p* < 0.05 were taken as statistically significant.

## 3. Results

### 3.1. Chromosome Gains in ALL-Derived Samples

Microarrays of CGHs were performed in two ALL-derived cell lines (JURKAT and CEM) and in 10 samples derived from the bone marrow of ALL patients to determine chromosome gains. As described in Table 2, the patients’ ages in the study ranged from 16 to 77 years, and most were male (7 out of 10). All the analyzed samples showed gains in at least two chromosomes; the most frequent chromosome gain was found for chromosome 17 (8 out of 12), followed by chromosomes 14 and 22 (6 out of 12).

### 3.2. Common Chromosome Gains in ALL-Derived Samples

**Figure 1:**
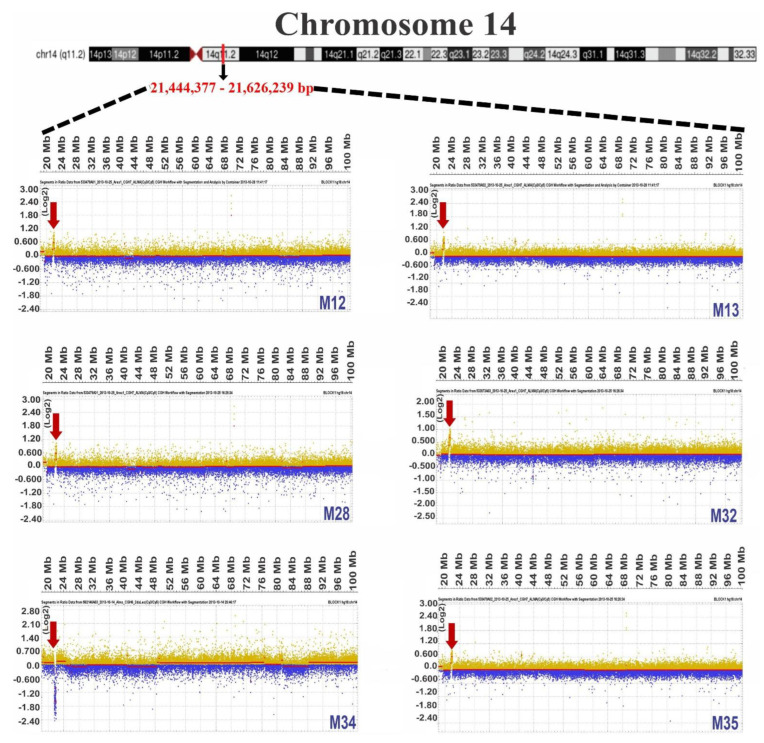
Comparative genomic hybridization images of gains observed for chromosome 14. The ideogram for chromosome 14 shows the common gain region on the q11.2 arm with dotted lines. Representative images of each patient or cell line were obtained with DEVA software; gains are indicated by red arrows, and signal intensity values (ratios) were normalized to Log2. The size scale is shown in megabases (Mb). The patient ID is included in the lower-right margin of each image.

**Figure 2:**
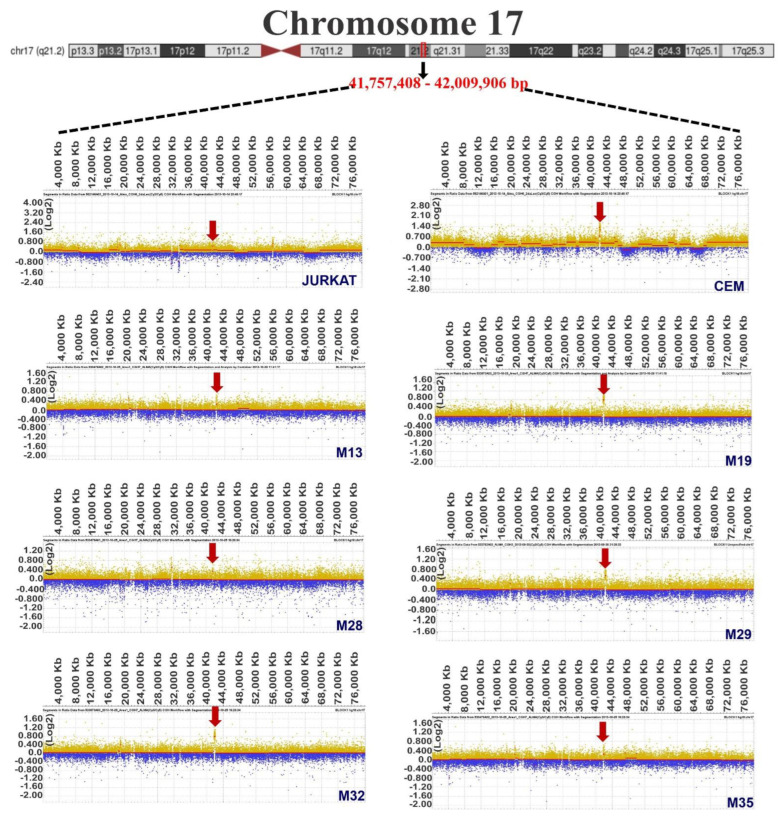
Comparative genomic hybridization images of gains observed for chromosome 17. The ideogram of chromosome 17 shows the common gain region on the q21.2 arm with dotted lines. Representative images of each patient or cell line were obtained with DEVA software; gains are indicated by red arrows, and signal intensity values (ratios) were normalized with Log2. The size scale is shown in megabases (Mb). The patient ID is included in the lower-right margin of each image.

**Figure 3:**
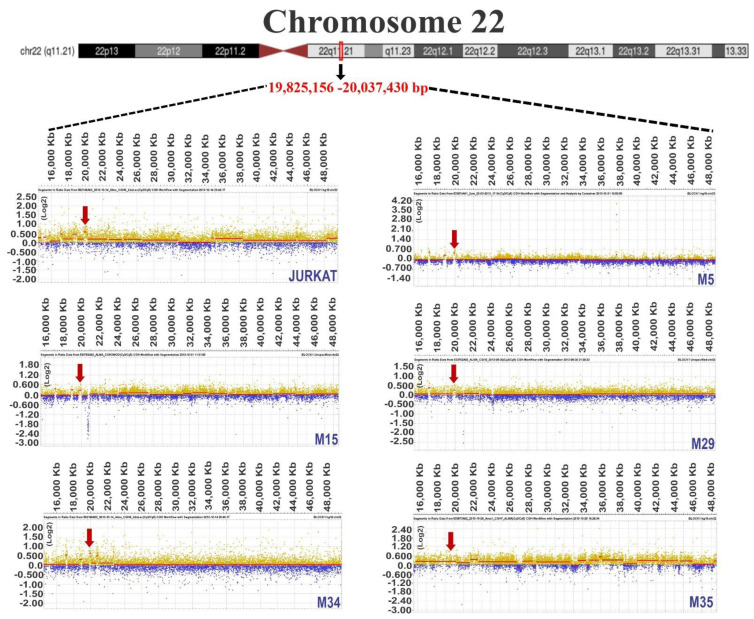
Comparative genomic hybridization images of gains observed for chromosome 22. The ideogram for chromosome 22 shows the common gain region on the q11.21 arm with dashed lines. Representative images of each patient or cell line were obtained with DEVA software; gains are indicated by red arrows, and signal intensity values (ratios) were normalized to Log2. The size scale is shown in megabases (Mb). The patient ID is included in the lower-right margin of each image.

Once it was determined that chromosomes 14, 17, and 22 contained the most frequent gains, we continued to determine each patient’s gain regions. After the regions were determined, we identified regions that overlapped in at least six samples, a condition fulfilled only for chromosomes 14, 17, and 22. Table 3 shows the start and end positions of the gains in each of the samples, as well as the sizes of the altered regions. Additionally, images obtained with *DEVA v 1.2* of the common gain zones in each sample are visualized in Figure 1, Figure 2 and Figure 3 for chromosomes 14, 17, and 22, respectively, including an ideogram containing the specific common gain regions.

**Figure 4:**
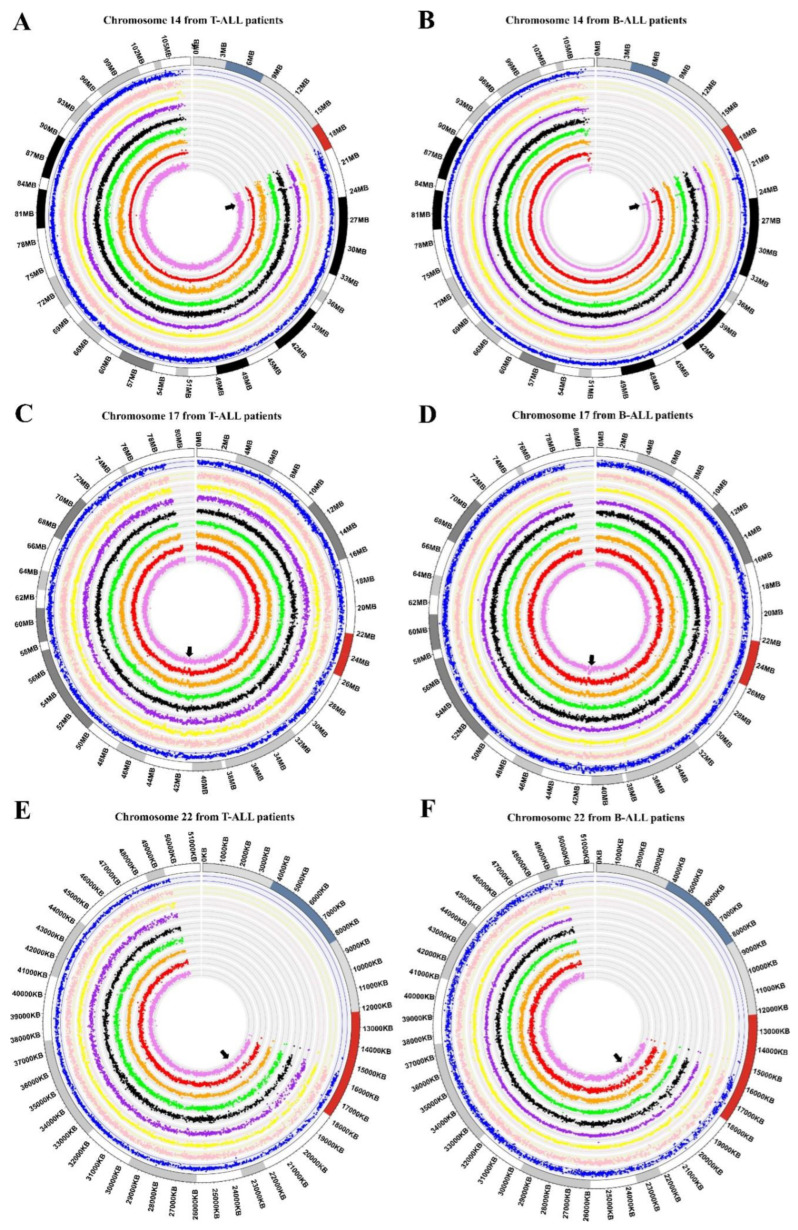
Circular multitrack plot representation of genomic data. The circos plots show ideograms of Log2-normalized genomic data from chromosome 14 (**A**,**B**), chromosome 17 (**C**,**D**), and chromosome 22 (**E**,**F**). Each level of the diagram represents a patient, and common zones of gain are highlighted with black arrows. As appropriate, the chromosome size scale is shown in megabases (Mb) or kilobases (Kb); (**A**,**C**,**E**) represent data from T-ALL; (**B**,**D**,**F**) represent data from B-ALL.

To improve the certainty of our results, we analyzed data from the GSE75671 study, which contained CGH arrays of bone marrow from adult patients with ALL and was performed with the same platform and Genome CGH arrays used in our study. From the 18 samples analyzed (nine T-ALL and nine B-ALL), eight and seven out of nine presented gain regions in T-ALL and B-ALL patients, respectively, for chromosome 14. Regarding chromosome 17, we identified nine (T-ALL) and five (B-ALL) samples and, in chromosome 22, four and five, respectively (as visualized in Figure 4). Normalized Log2 values for each patient are shown in Supplementary Table S2.

### 3.3. Identification of Genes Located in Regions of Chromosomal Gain

To identify genes present in chromosomal gain regions, we utilized UCSC Genome Browser (version GRch38/hg 38), and the following six genes were recognized for chromosome 14: *TOX4*, *METTL3*, *RAB2B*, *SALL2*, *OR10G3*, and *TRAV1-1.* For chromosome 17, the common gain region was determined to include 10 genes: *FKBP10*, *P3H4*, *DNAJC7*, *NT5C3B*, *CNP*, *ACLY*, *JUP*, *KLHL11*, *KLHL10*, and *TTC25-ODAD4*. Finally, six genes were found in the common gain zone established for chromosome 22: *RTL10-C22orf29*, *TXNRD2*, *COMT*, *ARVCF*, *GNB1L*, and *TANGO2*. All these genes can be visualized in Supplementary Figures S1–S3. In addition, the characteristics of each gene are described in Supplementary Table S1, including gene symbol, official full name, function, relationship with any kind of cancer, and reported gene alteration.

### 3.4. Expressions at mRNA Level of 22 Genes in JURKAT, CEM, and SUP-B15 Cell Lines

**Figure 5:**
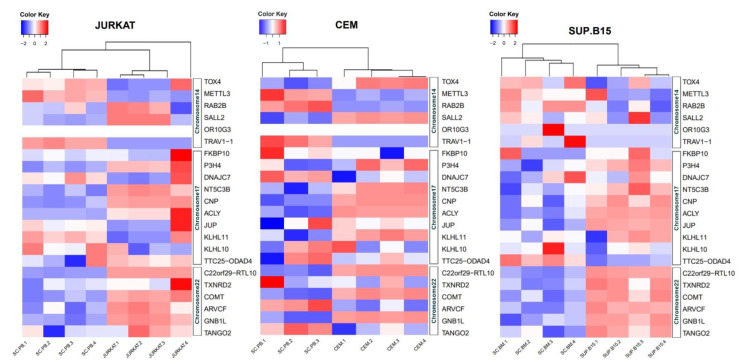
Normalized expression heatmaps (FPKM) of genes present in gain regions of chromosomes 14, 17, and 22 in the JURKAT, CEM, and SUP-B15 cell lines. The dendrogram depicts groups according to expression similarities. The samples included are: SC-PB—sample controls from peripheral blood; and SC-BM—sample controls from bone marrow. Data 1–4 in each of the cell lines represent independent sequencing; JURKAT4 and CEM4 were derived from our RNA sequencing (GSE189641).

Derived from the RNAseq analysis, heatmaps were created from JURKAT (T-ALL), CEM (T-ALL), and SUP-B-15 (B-ALL) cell lines versus nonleukemia controls (detailed in Materials and Methods). As observed in Figure 5, the *P3H4*, *NT5C3B*, *CNP*, *ACLY*, *RTL10*, *COMT*, and *GNB1L* genes maintained overexpression in the three cell lines, regardless of subtype. Interestingly, *JUP* mainly had a high expression in SUP-B15, while *SALL2* mainly had a high expression in JURKAT and CEM. For data validation, *JUP* expression was further determined by qPCR; as shown in Supplementary Figure S4, *JUP* expression was very high in SUP-B15 cells (changes of 46.9-fold taking *RPS18* as reference gene and 28.7-fold taking *RPLP0*), while in CEM and JURKAT, it was almost undetectable.

### 3.5. Expressions of 22 Genes in ALL Patients

**Figure 6:**
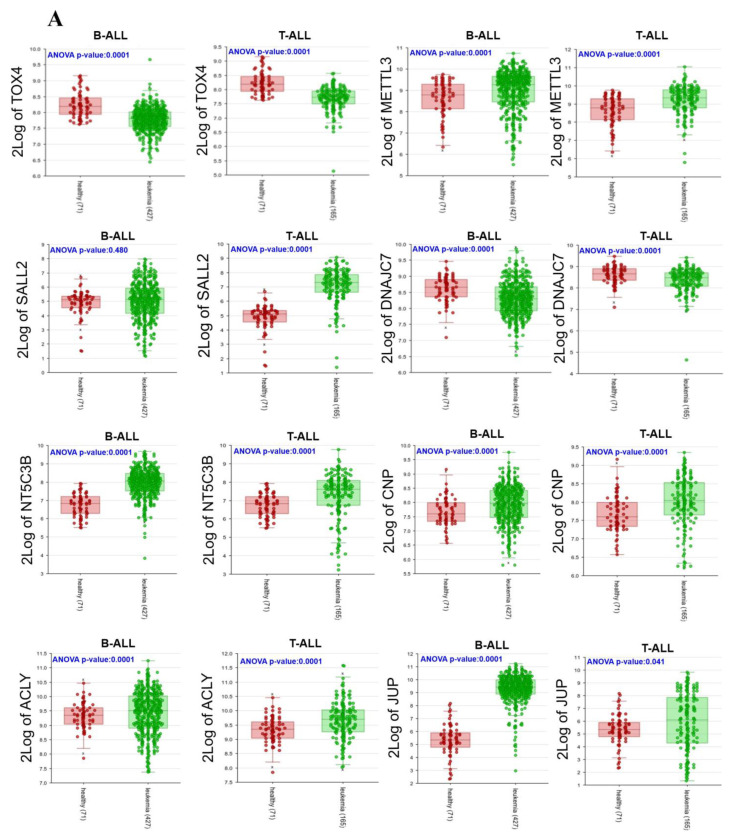

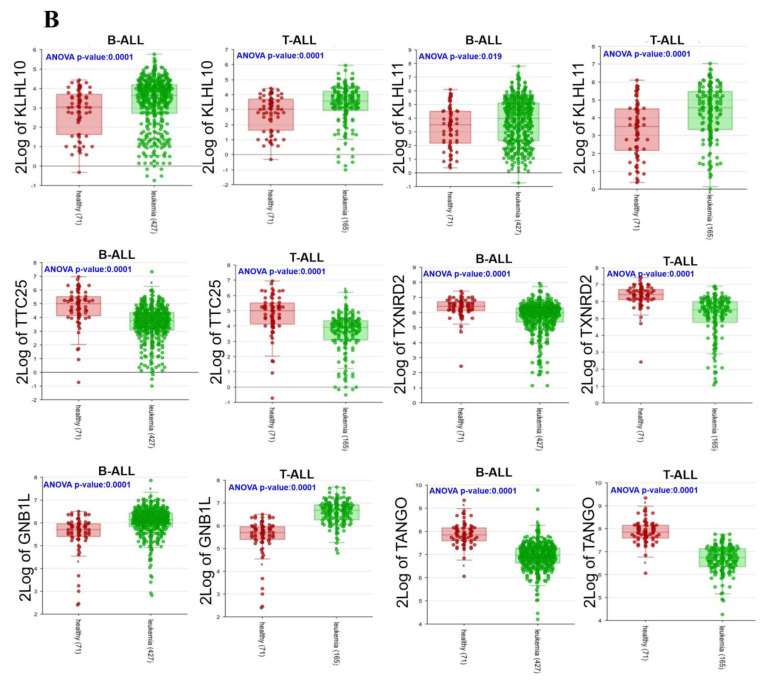
(**A**,**B**) Expressions of gain region genes in healthy individuals compared with ALL patients. Analysis of each gene was performed using the MILE project dataset. Boxplots show expressions at the RNA level in B-ALL or T-ALL subtypes, including ANOVA *p*-values. Numbers shown in parentheses correspond to the number of individuals analyzed for each gene. 2Log: gene expression transformed into logarithm with base 2.

The relative expression at the RNA level of each gene was determined using accessible microarray expression databases from the MILE project, as described in Materials and Methods. As observed in Figure 6A,B, seven genes showed statistically significant higher expressions in B-ALL and T-ALL patients compared with healthy individuals (*METTL3*, *NT5C3B*, *CNP*, *JUP*, *KLHL10*, *KLHL11*, and *GNB1L*), while *SALL2* and *ACLY* were overexpressed only in T-ALL patients. The gene that was found to be most significantly overexpressed in B-ALL was *JUP*. Unexpectedly, *TOX4*, *DNAJC7*, *TTC25/ODAD4*, *TXNRD2*, and *TANGO2* were underexpressed in ALL patients. No significant differences were found in other genes.

### 3.6. Hierarchical Trees in Normal Hematopoiesis and Different Leukemia Lineages of the Gain Genes

**Figure 7:**
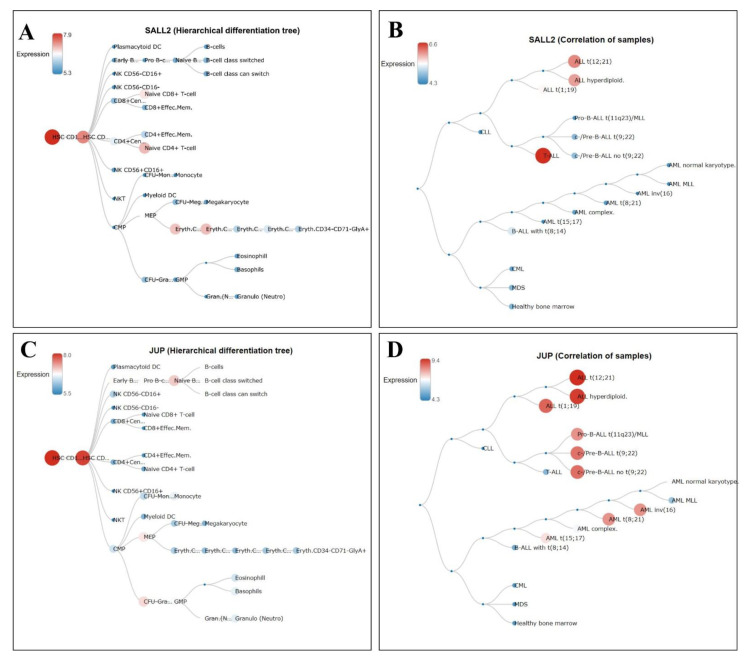
Hierarchical trees of *SALL2* and *JUP* using BloodSpot. Expressions of *SALL2* (**A**) and *JUP* (**C**) during the hematopoietic process. The expression scale is indicated in each graph from red (higher expression) to blue (lower expression). Full names are included from left to right: HSC-CD1—hematopoietic stem cell_CD133+CD34dim; HSC-CD—hematopoietic stem cell_CD38-_CD34+; DC—dendritic cell; CD8+Cen—CD8+Central memory; CD4+Cen—CD4+Central memory; CFU—colony-forming unit; NK—natural killer; CMP—common myeloid progenitor; MEP—megakaryocyte/erythroid progenitor; Eryth C—erythroid _CD34+CD71+GlyA-, erythroid _CD34-CD71+GlyA-, erythroid _CD34-CD71+GlyA+; Gra—granulocyte; GMP—granulocyte/monocyte progenitor; Gran. (N…—granulocyte neutrophilic metamyelocyte. Expressions of *SALL2* (**B**) and *JUP* (**D**) in different leukemia subtypes. CLL—chronic lymphoblastic leukemia; CML—chronic myeloid leukemia; MDS—myelodysplastic syndromes.

Once we identified the genes with statistically significant high expressions in B-ALL or T-ALL patients, we were interested to know the expression of each gene during normal hematopoiesis and to compare it with the expressions in all the leukemia lineages. Each gene was analyzed utilizing BloodSpot, as detailed in Materials and Methods. After evaluation, we determined that evident differences were observed just in *SALL2* and *JUP* since, during normal hematopoiesis, both genes were highly expressed only in hematopoietic stem cells (HSCs) (Figure 7A,C, respectively); this was a relevant difference between healthy bone marrow and the leukemia lineages. As shown in Figure 7B, *SALL2* was strongly expressed principally in T-ALL samples, followed by ALL t(12;21) and ALL hyperdiploid samples. However, *JUP* was principally found to be highly expressed in ALL t(12;21), ALL hyperdiploid, and ALL t(1;19), followed by Pro- and Pre-B-derived samples. In addition, moderate expressions were observed in some AML subtypes, as seen in Figure 7D.

### 3.7. Relationship between Highly Expressed Genes and ALL Patients with Poor Survival

**Figure 8:**
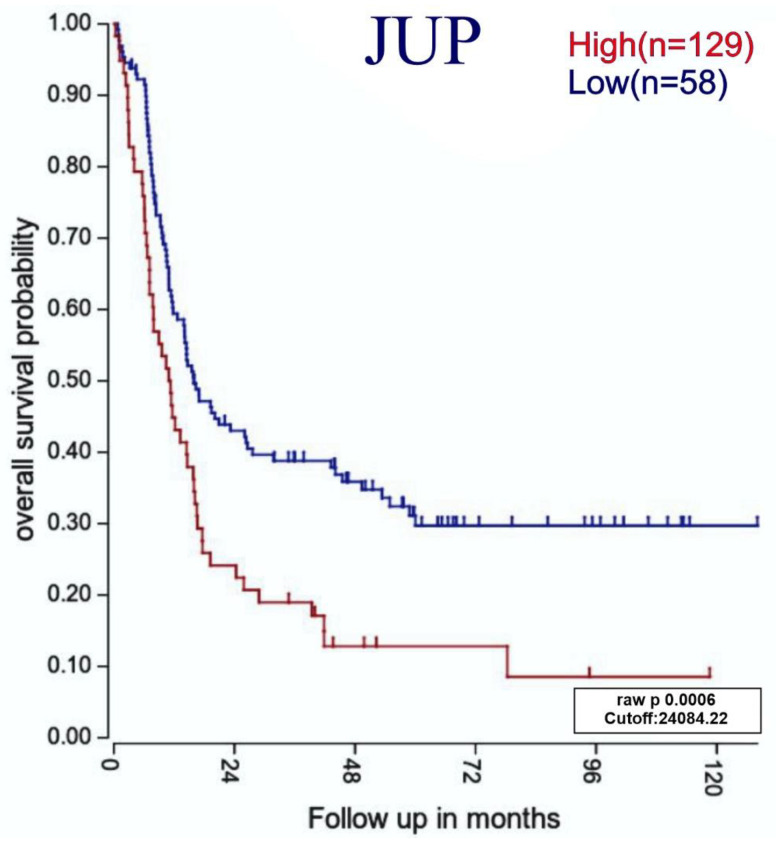
Overall survival probability analysis of *JUP* using data derived from microarray expressions of adults with B-ALL. Kaplan–Meier survival curves represent the relationship of the probability of survival over time (measured in months) divided into high- and low-expressing groups. The cutoff point was calculated according to expression level (raw) for separate individuals with high (red) and low (blue) gene expressions.

Since we saw relevant differences in the *SALL2* and *JUP* expressions in leukemia patients in contrast to healthy bone marrow samples, we determined whether the expressions of these genes were related to better overall survival. Since survival studies require a long follow-up period and we do not yet possess these data in our working group, we decided to search for a free database containing this information. After an exhaustive search, only one free database on the follow-up of adult B-ALL patients was found (as described in Materials and Methods). After Kaplan–Meier curve analysis, as shown in Figure 8, the high expression of *JUP* was statistically significantly associated with poorer overall survival.

## 4. Discussion

Acute lymphoblastic leukemia (ALL) involves the disruption of differentiation in a clonal lymphoid population in the early stages [11] that can invade the bone marrow, blood, and extramedullary sites [49]. ALL is mainly a genetic disease because most patients present chromosomal alterations, and the characterization of these anomalies has become a valuable tool in clinical settings since some genes are used as prognostic and diagnostic markers [50], or even therapeutic targets [14,51]. Thus, this study aimed to determine the genes present in the most frequent gain regions in cell lines and cells derived from ALL patients by employing CGH microarrays. We found that the most affected chromosomes in at least 50 percent of the analyzed samples were 14, 17, and 22 (Table 2). Usvasalo et al. reported frequent increase in the number of copies in chromosomes 1, 5, 8, 10, 14, and 21 in patients with ALL [52]. In addition, assays performed in other studies agree with our findings because they also show gains in chromosomes 14, 17, and 22 [53,54,55]. These results were consistent when we contrasted the altered gain regions of the three chromosomes with CGH data (GSE75671) from 18 patients with ALL, as seen in Figure 4 and Supplementary Table S2 [31].

Regarding the results visualized in Table 2, it is noteworthy to mention that, just by looking at the gains, some patients had alterations in more than half of their chromosomes. The majority of malignant diseases have some underlying form of instability; chromosomal instability (CIN) is one of the characteristics of cancer, and it includes the loss or amplification of driver genes, focal rearrangements, extrachromosomal DNA, micronuclei formation, and activation of innate immune signaling [56], among others, which can drive phenotypic adaptation during tumor evolution [57].

In this study, it was of our interest not only to identify gains in genes, but also to validate their expressions at the mRNA level. As expected, we found genes that had already been widely reported, which validated our study. However, the originality of this work is that we identified genes that had not been previously linked to leukemia or any other cancer type, as seen in Supplementary Table S1. Of the 22 genes identified in the chromosomal gain regions, those that maintained high expressions in leukemia-derived cell lines and in ALL patients (Figure 5 and Figure 6A,B) were *NT5C3B*, *CNP*, *ACLY*, *JUP*, *KLHL11*, *RTL10*, and *GNB1L*.

There is limited information about two of the genes mentioned above: *NT5C3B* (5′-nucleotidase, cytosolic IIIB), which has until now only been involved in processes of the respiratory tract and atherosclerosis [58,59], and *KLHL11* (Kelch-like family, member 11) [60], part of the Kelch family, which has only been related to paraneoplastic encephalitis with an oncological profile [61]. To date, neither of these genes has been associated with leukemia or other types of cancer; therefore, delving into the mechanisms of these genes in the context of leukemia is of great importance and provides a new research perspective.

Regarding *CNP*, the enzyme 2′,3′-cyclic nucleotide 3′ phosphodiesterase [62], it has until now only been linked to glioblastoma multiforme (GBM); *CNP*-positive patients had better survival rates than individuals with *CNP*-negative tumors [63]. Although we focused on showing genes whose high expressions had poor prognoses in leukemia, it is important to mention that these results were consistent with our findings in B-ALL, since high *CNP* expression correlated with better overall survival (data not shown).

Concerning *ACLY*, which translates the enzyme ATP citrate lyase and is responsible for the synthesis of cytosolic acetyl-CoA [64], an association with ALL has also not been reported; however, in AML, there was evidence that patients with low levels of this gene had favorable prognoses [65]. In other types of cancer, *ACLY* upregulation has been shown to promote metastasis and invasion and to inhibit apoptosis in prostate [66], colon [67], breast [68], and esophageal cancer cells [69]. Specifically, it was proposed as a predictive and recurrent biomarker in breast cancer [68]. In contrast, the opposite was observed when we analyzed the survival curves in B-ALL samples (data not shown).

In relation to *JUP/Plakoglobin*, a gene that produces the protein γ-catenin and is homologous to β-catenin [70], it was reported to be necessary for maintaining the “*BCR-ABL1*” genetic abnormality (through regulation of *MYC* and *BIRC5*/survivin) in B-ALL, being proposed as a potential therapeutic target [71]. In addition, γ- and β-catenin were essential for maintaining leukemic stem cells in AML [72], while γ-catenin has also been found to be overexpressed in ovarian [73] and gastric cancer [74]. *JUP* expression in prostate cancer is controversial since changes depending on the stage [75]. In our study, *JUP* was found to be highly expressed in the SUP-B15 cell line and B-ALL individuals (Figure 5 and Figure 6A, respectively); moreover, its high expression yielded a significant correlation with worse overall survival in B-ALL (Figure 8). It is essential to highlight that our results support its great utility in clinical settings, not only as a prognostic marker, but also as a therapeutic target since, additionally, the hierarchical tree analysis demonstrated its high expression only in HSC during normal hematopoiesis and in diverse B-ALL phenotypes (Figure 7).

Another gene that we found to have a high expression was *RTL10*, also known as *C22orf29* (retrotransposon gag-like 10). This gene translates to a BH3 protein, a motif that is part of the proapoptotic proteins Bad, Bik, and PUMA. The role of the BH3 protein in ALL, individually or as a motif of proapoptotic proteins, has not been described; nevertheless, PUMA levels were elevated in chronic lymphoblastic leukemia (CLL) [76]. These data correlate with our findings in ALL; however, no correlation was found between its expression and survival (data not shown).

In our study, we found overexpression of *GNB1L* (G protein beta 1 subunit) in the cell lines and ALL patients; however, there are no reports in the literature that have studied the expression of this gene in any type of cancer. Most studies on *GNB1L* are related to psychiatric disorders reported, for example, in schizophrenia [77] and autism [78]. Only one report exists in which alterations in copy number variations (CNVs) were found in hepatocellular carcinomas, but only in 18 out of 98 patients [79]; thus, we considered this gene as another prominent candidate for further functional studies in ALL.

Concerning *SALL2* (spalt-like transcription factor 2), which is a member of the spalt/sal family of transcription factors associated with cell differentiation, development, and stemness [80], its expression in cancer is still controversial. In a transcriptome and genome analysis performed for children and adults with ALL, the fusion of *SALL2* with *TCR-α* (*TRA-SALL2*) was one of the most recurrent fusions reported in this pathology [81]. In addition, high expressions of *SALL2* have been found in esophageal cancer [82], breast cancer [83], testicular cancer [84], and glioblastoma [85]. In contrast, losses or reduced expressions have been reported in HL-60 and primary acute myeloid leukemia samples [86], as well as in ovarian [87] and oral cancer [88]. Since we found that *SALL2* was overexpressed in T-ALL-derived cells and because, during normal hematopoiesis, its expression is limited to HSCs, we believe that the expression of this gene could be useful as a lineage marker and as a potential therapeutic target.

On the other hand, there were two genes in which overexpression was determined in leukemia-derived cell lines but was not confirmed in patients: *COMT* and *P3H4* (Figure 4). With respect to *COMT*, this gene translates catechol-O-methyltransferase, an enzyme involved in the metabolic degradation of catecholamines, which normally exhibit low activity in leukocytes [89]; in pediatric ALL patients, the *COMT*-“rs4680” polymorphism was associated with mercaptopurine-induced hepatotoxicity [90]. In endometrial (rs4680) [91] and breast cancer (val158 met) [92], some polymorphisms of *COMT* have been evaluated; however, no significant associations have been found. In contrast, as we observed in the leukemia-derived cell lines, Hashimoto et al. found that *COMT* had low levels in patients and cell lines of prostate cancer; moreover, the restoration of its expression in DuPro and DU145 led to the suppression of migration and an increase in apoptosis [93]. Regarding *P3H4* (prolyl 3-hydroxylase family member 4), no association has been described with any type of leukemia; however, in lung and bladder cancer, the upregulation of mRNA and protein levels have been associated with the promotion of proliferation, migration, and invasiveness [94,95]. On the contrary, in kidney cancer, *P3H4* helped reduce cell invasion through miR-133a and miR-1a [96].

In addition, we found two genes statistically significantly overexpressed in B- and T-ALL patients but not in the cell lines: *METTL3* (N6-adenosine-methyltransferase) and *KLHL10* (Kelch-like family member 10). The former methylates primary microRNAs (pri-miRNAs) that promote the initiation of miRNA biogenesis [97]; alteration in their function has been related to promoting tumorigenicity, and high expressions have been observed in cervical [98], colorectal [99], prostate [100], pancreatic [101], ovarian [102], and esophageal cancer [103]. Furthermore, its overexpressions in bladder [104] and gastric cancer [105] have been associated with poor prognoses. Interestingly, it was reported that *METTL3* mRNA and protein expression was increased in acute myeloid leukemia (AML) cells compared to healthy hematopoietic stem and progenitor cells [106], and a higher expression was reported in pediatric ALL *ETV6/RUNX1*-positive patients when compared to controls [107]. Based on these observations, it has been proposed that the downregulation of this gene could be a therapeutic strategy [108,109]. Regarding *KLHL10*, there is limited information about its function, and there are no studies that associate it with cancer or any other pathology; to date, it has been related only to spermatogenesis and male infertility [110,111], and this is the first time that this gene has been associated with ALL. Therefore, we consider that this gene should also be functionally studied in the context of this pathology.

Unexpectedly, although our study observed gains in genomic DNA (Figure 1, Figure 2 and Figure 3), lower expressions in ALL patients compared to healthy individuals were observed in *TOX4*, *DNAJC7*, *TTC25*, *TXNRD2*, and *TANGO2* (Figure 6A,B). Regarding TOX4, it was reported that this gene was highly expressed in AML patients compared with clinically healthy individuals [112]. In other types of cancer, such as breast and lung cancer, it was determined that TOX4 was highly expressed [113]; however, it has not yet been linked to LLA. No association with the expression of DNAJC7 (DnaJ heat shock protein family (Hsp40) member C7) has been reported; however, the accumulation in serum of *DNAJC*7 was proposed as a potential biomarker in renal cell carcinoma early detection [114]. In the case of *TTC25*, also known as *ODAD4* (outer dynein arm docking complex subunit 4), it is characterized by its participation in mucociliary clearance [115]. Interestingly, in a study where researchers stimulated PBMCs in horses with LPS, overexpression of this gene was found at the level of the transcriptome, but the reasons were unknown [116]. Regarding thioredoxin reductase 2 (*TXNRD2/TRXR2*), an important antioxidant enzyme that controls the levels of cellular reactive oxygen species (ROS), it was identified as being hypomethylated in CLL [117]. Furthermore, higher levels of this gene have been observed in hepatocellular carcinomic tissues [118], as well as in NSCLC (non-small-cell lung carcinoma) [119]. Concerning *TANGO2* (transport and organization homolog of the golgi complex 2), polymorphism in “p. Ser17Ter” was associated with an aggressive profile of prostate cancer [120]. According to the previously mentioned reports, overexpressions of these genes would be expected at the transcriptome level. Further studies using other methodologies are necessary to determine their roles in leukemia.

## 5. Conclusions

This study identified genes present in common chromosomal gains detected through CGH microarrays in ALL patients and two classical leukemia-derived cell lines and validated them using expression microarrays and RNAseq. The expressing genes that we thought could be clinically relevant were *SALL2*, *NT5C3B*, *CNP*, *ACLY*, *JUP*, *KLHL11*, *RTL10*, and *GNB1L*. Of these, it is worth mentioning that a hierarchical tree analysis showed very high expressions of *SALL2* in T-ALL and *JUP* in B-ALL patients, as well as in HSC, but not in any other cell lineage generated during normal hematopoiesis. Moreover, it is essential to highlight that the overexpression of *JUP* was strongly related to poor overall survival in B-ALL patients. Deepening the study of these genes are relevant constitutes a fertile field of research to investigate how their alterations are related to this pathology and to evaluate with more sensitive tests their application in the diagnosis and prognosis of ALL, as well as their utility as therapeutic targets.

## Figures and Tables

**Table 1:** Primer sequences used to evaluate *JUP* expression by qPCR.

Gene	Forward	Reverse	Size amplicon
***JUP***	AGCAGCCCTACACGGATG	GATGTTCTCCACCGACGAGTA	161 bp
***RPS18***	CGATGGGCGGCGGAAAA	CAGTCGCTCCAGGTCTTCACGG	283 bp
***RPLP0***	CCTCATATCCGGGGGAATGTG	GCAGCAGCTGGCACCTTATTG	100 pb

**Table 2:** Description of chromosomes that presented gain alterations in patients with ALL, including JURKAT and CEM leukemia-derived cell lines.

Sample	Sex	Age	Type of Leukemia	Chromosome with Gains
**JURKAT**	M	14	T-ALL	1,3,5,6,11,13,15,17,22
**CEM**	F	4	T-ALL	5,8,11,12,13,14,15,16,17,18,19,20,21,X
**M5**	F	49	Pre-B-ALL	3,5,7,9,10,11,12,13,14,15,16,17,18,19,20,22
**M12**	M	77	T-ALL-T	1,14,17
**M13**	M	65	T-ALL	7,14,17
**M15**	M	17	Pre-B-ALL	5,14,15,19,21,22
**M19**	M	45	Pre-B-ALL	14,17
**M28**	M	16	B-LLA	4,14,17,Y
**M29**	F	50	B-LLA	1,6,8,10,14,15,17,18,19,22,X
**M32**	F	31	T-LLA	14,17
**M34**	M	20	B-LLA	1,12,14,15,16,17,18,19,22,X
**M35**	M	16	B-LLA	7,9,10,12,14,15,17,19,22,Y

**Table 3:** Description of chromosomal gains found in each of the samples analyzed. Chr: chromosome; Pb: base pairs.

Sample ID	Chr	Position of Gain Pb	Size (Pb)
**JURKAT**	17	41,757,408–42,009,906	252,498
	22	14,507,171–30,395,708	15,888,537
**CEM**	17	36,734,811–46,367,425	9,632,614
**M5**	22	19,825,156–20,037,430	212,274
**M12**	14	21,444,377–21,624,239	179,862
**M13**	14	21,441,167–21,723,152	281,985
	17	41,755,952–42,135,245	379,293
**M15**	22	19,718,309–20,037,430	319,121
**M19**	17	35,353,354–42,945,517	7,592,163
**M28**	14	21,441,167–22,013,159	571,992
	17	40,964,001–43,704,741	2,740,740
**M29**	17	41,307,415–43,247,247	1,939,832
	22	19,720,528–20,037,430	316,902
**M32**	14	21,424,744–21,961,837	537,093
	17	40,741,630–43,408,899	2,667,269
**M34**	14	21,444,229–22,055,727	611,498
	22	19,797,537–20,037,970	240,433
**M35**	14	21,441,167–22,046,172	605,005
	17	41,526,491–42,128,942	602,451
	22	15,567,273–26,691,944	11,124,671

## Data Availability

The CGH microarray raw data presented in this study are openly available in the Gene Expression Omnibus (GEO) database repository (https://www.ncbi.nlm.nih.gov/geo/query/acc.cgi accessed on 31 October 2021) under GEO accession number GSE185274.

## Supplementary Materials

The following supporting information can be downloaded at: https://www.mdpi.com/article/10.3390/diagnostics12112788/s1, Supplementary Figures S1–S3 show genes present in common gain regions visualized with UCSC Genome Browser on Humans (GRCh38 vs. hg38) for chromosomes 14, 17, and 22, respectively. Supplementary Figure S4 shows relative expressions of *JUP* in peripheral blood and leukemia-derived cell lines. Supplementary Table S1 presents the main features of 22 genes found with gain, and Supplementary Table S2 shows data of DNA copy numbers normalized to Log2 for chromosomes 14, 17, and 22 using GSE75671 data.
