# Supplementary material for: Comparative Genomic Hybridization and Transcriptome Sequencing Reveal Genes with Gain in Acute Lymphoblastic Leukemia: JUP Expression Emerges as a Survival-Related Gene
Source: Diagnostics (Basel). 2022 Nov 14;12(11):2788. doi: 10.3390/diagnostics12112788 (PMC9689318; doi:10.3390/diagnostics12112788)
Supplement: Supplementary file 1 [file Supplementary Figure S3.pdf › Supplementary Figure S3.pdf]

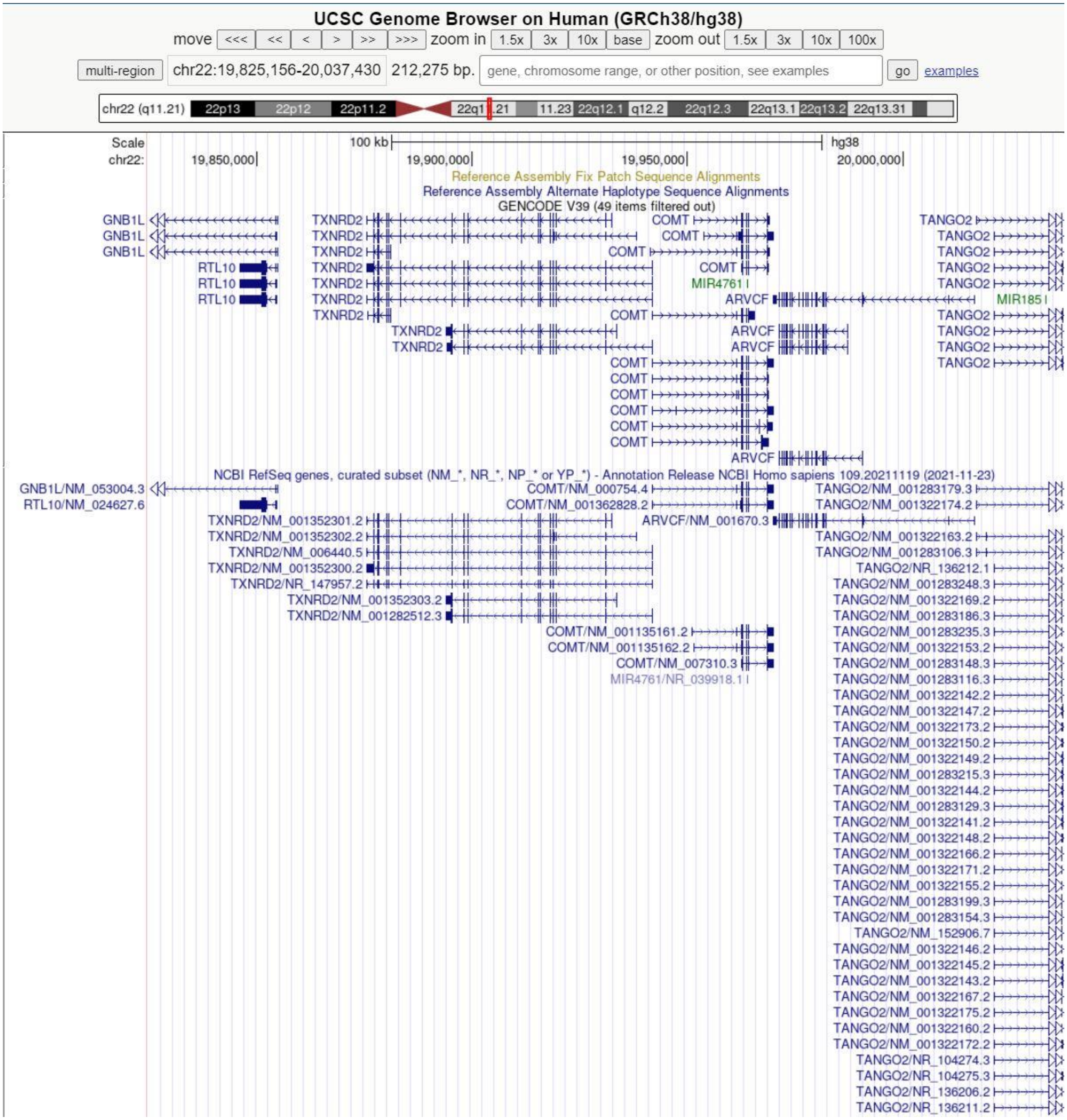

Supplementary Figure S3. Genes present on the common gain region of chromosome 22, found between 19,825,156 to 20,037,430 base pairs of the genome, visualized with the UCSC Genome Browser. The six genes in blue are coding products with their respective isoforms, and in green are non-coding transcripts.
