# Supplementary material for: Comparative Genomic Hybridization and Transcriptome Sequencing Reveal Genes with Gain in Acute Lymphoblastic Leukemia: JUP Expression Emerges as a Survival-Related Gene
Source: Diagnostics (Basel). 2022 Nov 14;12(11):2788. doi: 10.3390/diagnostics12112788 (PMC9689318; doi:10.3390/diagnostics12112788)
Supplement: Supplementary file 1 [file diagnostics-12-02788-s001.zip › Supplementary Figure S2.pdf]

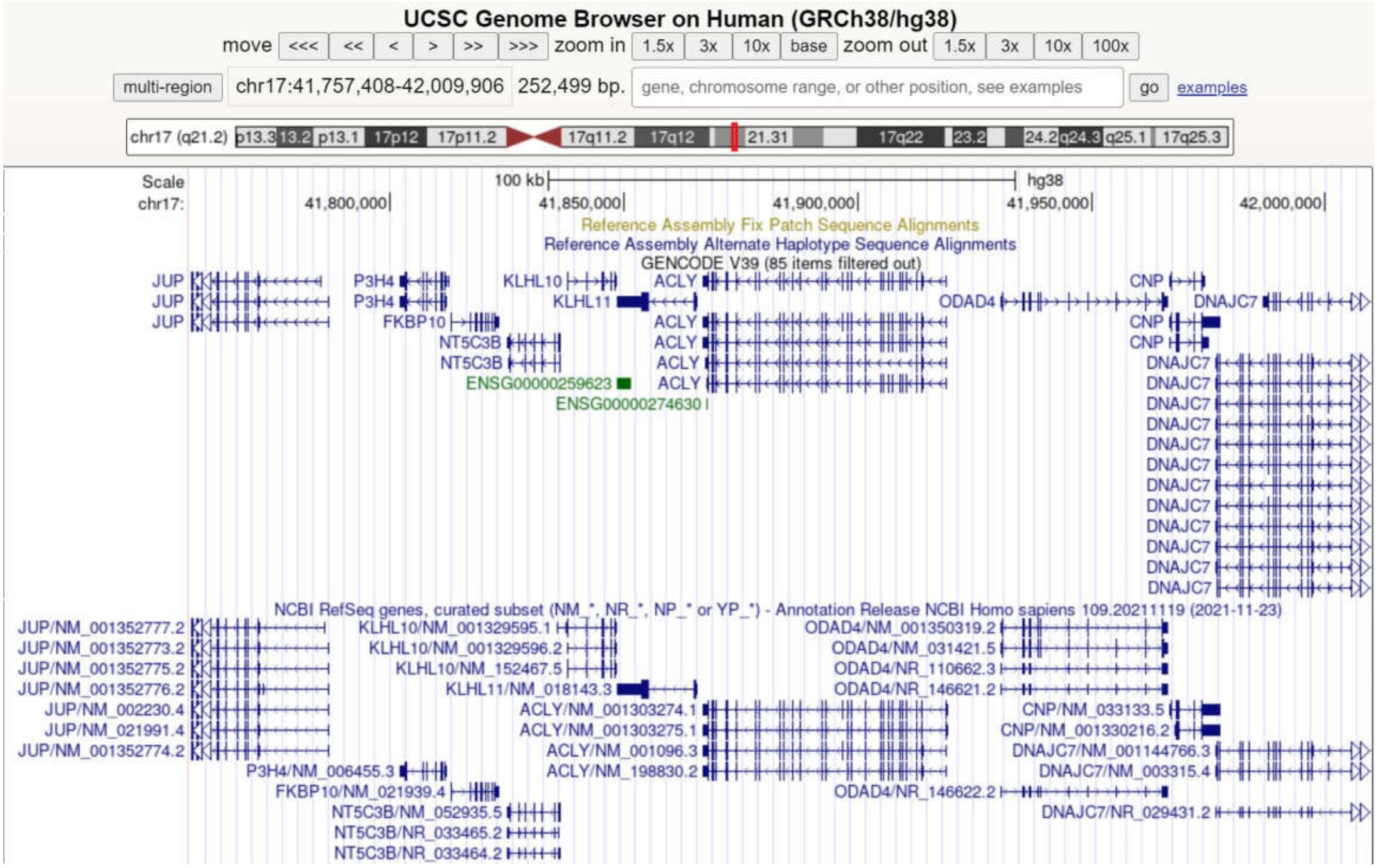

Supplementary Figure S2. Genes present on the common gain region of chromosome 17, found between 41,757,408 to 42,009,906 base pairs of the genome, visualized with the UCSC Genome Browser. The ten genes in blue are coding products with their respective isoforms, and in green are non-coding transcripts.
