# Supplementary material for: Comparative Genomic Hybridization and Transcriptome Sequencing Reveal Genes with Gain in Acute Lymphoblastic Leukemia: JUP Expression Emerges as a Survival-Related Gene
Source: Diagnostics (Basel). 2022 Nov 14;12(11):2788. doi: 10.3390/diagnostics12112788 (PMC9689318; doi:10.3390/diagnostics12112788)
Supplement: Supplementary file 1 [file diagnostics-12-02788-s001.zip › Supplementary Figure S4.pdf]

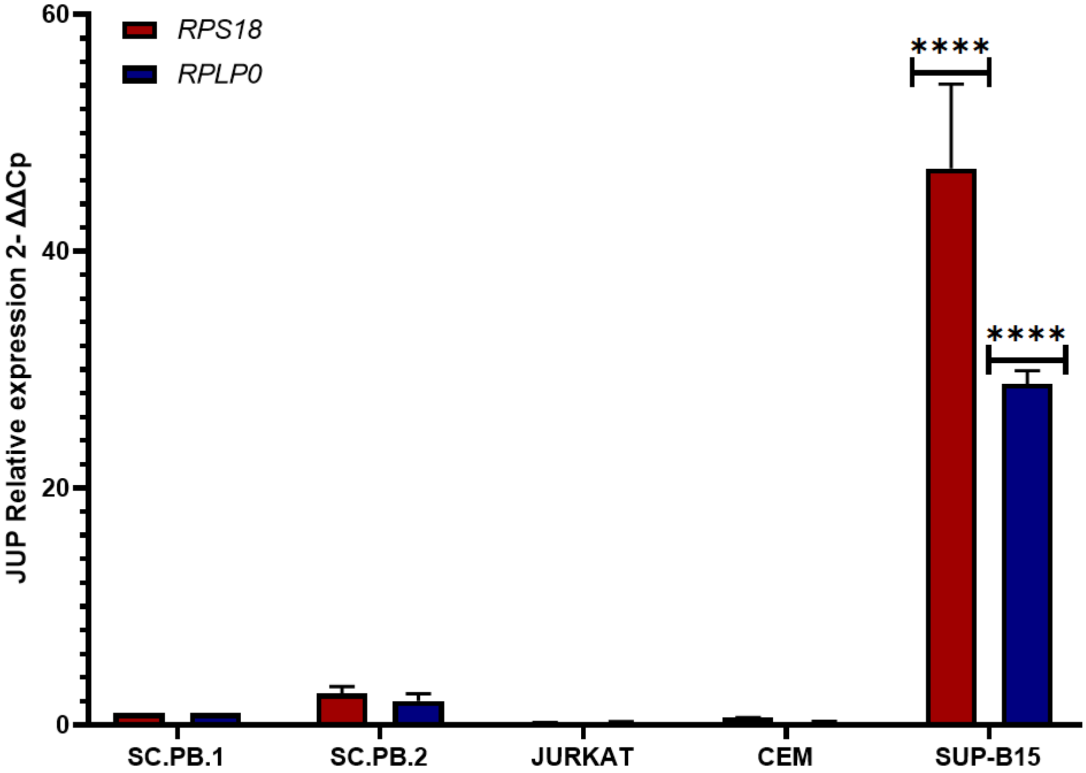

	SC.PB.1	SC.PB.2	JURKAT	CEM	SUP-B15
RPS18	1.0	2.65+/- 0.58	0.19+/- 0.03	0.60+/- 0.04	46.97+/- 7.13
RPLP0	1.0	2.0+/- 0.61	0.27+/- 0.02	0.045+/- 0.02	28.79+/- 1.1

Supplementary Figure S4. Relative expression of *JUP* in peripheral blood and leukemia-derived cell lines. qPCR assays were used to determine mRNA levels tacking *RPLP0* and *RPS18* as reference genes. SC.PB.1 and SC.PB.2 are sample controls; the first was set as calibrator. The difference between all groups was calculated with a one-way ANOVA test. Asterisks indicate statistical significance ($p < 0.0001$). An average and standard deviation of the Fold change obtained with the *RPS18* and *RPLP0* reference genes are depicted.
