# Supplementary material for: Comparative Genomic Hybridization and Transcriptome Sequencing Reveal Genes with Gain in Acute Lymphoblastic Leukemia: JUP Expression Emerges as a Survival-Related Gene
Source: Diagnostics (Basel). 2022 Nov 14;12(11):2788. doi: 10.3390/diagnostics12112788 (PMC9689318; doi:10.3390/diagnostics12112788)
Supplement: Supplementary file 1 [file diagnostics-12-02788-s001.zip › Supplementary Table S1.pdf]

Supplementary Table S1. Characteristics of the 22 genes found on chromosomes 14, 17, and 22.

Gene symbol	Official full name	Function	Relationship with any kind of cancer	Gene alteration and others
TOX4	TOX high mobility group box family member 4	*TOX4* is one of the regulatory factors of the PTW/PP1 phosphatase complex, which plays a role in the control of chromatin structure and cell cycle progression during the transition from mitosis into interphase.	Acute Mieloid Leukemia [1]	Overexpressed
			Breast cancer [2]	Overexpressed
			Lung cancer [2]	Underexpressed
			Kidney cancer [3]	Overexpressed
METTL3	Methyltransferase 3, N6-adenosine-methyltransferase complex catalytic subunit	The *METTL3-METTL14* heterodimer forms a N6-methyltransferase complex that methylates adenosine residues at the N(6) position of some RNAs and regulates various processes such as the circadian clock, differentiation of embryonic and hematopoietic stem cells, cortical neurogenesis, response to DNA damage, differentiation of T-cells and primary miRNA processing.	Acute myeloid leukemia [4,5]	Overexpressed
			Cervical cancer [6]	Overexpressed
			Colorectal cancer [7]	Overexpressed
			Bladder cancer [8]	Overexpressed
			Gastric cancer [9]	Overexpressed
			Prostate cancer [10]	Overexpressed
			Pancreatic cancer [11]	Overexpressed
			Ovarian cancer [12]	Overexpressed
			Esophageal cancer [13]	Overexpressed
			Gastrointestinal cancer [14]	Overexpressed
			Papillary thyroid cancer [15]	Underexpressed
RAB2B			Cervical cancer [16]	Overexpressed

Gene symbol	Official full name	Function	Relationship with any kind of cancer	Gene alteration and others
	RAB2B, member *RAS* oncogene family	*RAB2B* is a member of the Rab protein family are non-transforming monomeric GTP-binding proteins of the Ras superfamily that contain four highly conserved regions involved in GTP binding and hydrolysis. *RAB2B* is required for protein transport from the endoplasmic reticulum to the Golgi complex.	Pancreatic cancer [17]	Overexpressed
SALL2	Spalt like transcription factor 2	*SALL2* encodes a protein containing multiple zinc finger domains, which belongs to transcription factors (TFs), which are master regulators of gene expression and can control transcriptional production and, as a result, the proliferative or differentiated phenotype of the cell. *SALL2* has been mainly involved in optical fissure closure during the development of the eye in the embryo.	Acute lymphoblastic leukemia [18]	Frequent fusion-*TRA-SALL2*
			Acute myeloid leukemia [19]	Underexpressed
			Esophageal cancer [20]	Overexpressed
			Breast cancer [21]	Underexpressed
			Testicular cancer [22]	Overexpressed
			Oral Squamous Carcinomas [23]	Underexpressed
			Glioblastoma [24]	Overexpressed
			Ovarian carcinoma [25]	Underexpressed
OR10G3	Olfactory receptor family 10 subfamily G member 3	*OR10G3* belongs to the olfactory receptor proteins are members of a large family of G protein-coupled receptors (GPCRs) that arise from single-exon coding genes. Olfactory receptors are responsible for G protein-mediated recognition and transduction of odor signals.	None	None
TRAV1-1	Receptor de células T alfa variable 1-1	This gene forms part of the alpha chain of the TCR of lymphocytes.	Breast cancer [26]	High frequency

Gene symbol	Official full name	Function	Relationship with any kind of cancer	Gene alteration and others
FKBP10	FKBP prolil isomerasa 10	The protein encoded by this gene belongs to the FKBP-type peptidyl-prolyl cis/trans isomerase (PPIase) family. This protein localizes to the endoplasmic reticulum and acts as a molecular chaperone. It has also been identified as FKBP65 and has a high binding affinity for the immunosuppressant drugs cyclosporin A (CsA) and FK506.	Leukemia [27]	K562 cells-Adriamycin resistance
			Stomach cancer [28]	Overexpressed
			Gastric cancer [29]	Overexpressed
			Lung cancer [30]	Overexpressed
			Renal cancer [31]	Overexpressed
			Glioma [32]	Overexpressed
			Ovarian cancer [33]	Underexpressed
			Breast cancer [34]	Overexpressed
P3H4	prolyl 3-hydroxylase family member 4	*P3H4* translates the nucleolar protein first characterized as an autoantigen in cases of interstitial cystitis. Together with PLOD1, P3H3 forms a complex that catalyzes the hydroxylation of lysine residues in collagen alpha chains and is required to assemble and normally crosslink collagen fibrils. Required for normal bone density and normal skin stability through its role in the hydroxylation of lysine residues in collagen alpha chains and collagen fibril assembly.	Bladder cancer [35]	Overexpressed
			Lung cancer [36]	Overexpressed
			Renal cancer[37]	Underexpressed
DNAJC7	DnaJ heat shock protein family (Hsp40) member C7	This gene encodes a member of the DNAJ heat shock protein 40 family. This protein binds the chaperone heat shock proteins 70 and 90 in an ATP-dependent manner and may function as a co-chaperone. Pseudogenes of this gene are found on chromosomes 1 and 6.	Kidney cancer [38]	Overexpressed
NT5C3B	5'-nucleotidase, cytosolic IIIB	*NT5C3B* translates the enzyme 5'-nucleotidase, cytosolic IIIB, which catalyzes dephosphorylation of 7-methylguanosine	None	None

Gene symbol	Official full name	Function	Relationship with any kind of cancer	Gene alteration and others
		monophosphate (m7GMP) released during mRNA turnover. The specific activity for m(7)GMP may protect cells against undesired salvage of m(7)GMP and its incorporation into nucleic acids.		
CNP	2',3'-cyclic nucleotide 3' phosphodiesterase	*CNP* translates to enable 2',3'-cyclic-nucleotide 3'-phosphodiesterase activity. This CNPase is a membrane-bound enzyme found at high concentrations in central nervous system myelin and in the outer segments of photoreceptors in the retina that may participate in RNA metabolism in the myelinating cell. Two proteins with CNP activity exist in the brain and lymphoid tissues.	Glioblastoma [39]	Overexpressed
ACLY	ATP citrate lyase	In many tissues, ATP citrate lyase is the primary enzyme for synthesizing cytosolic acetyl-CoA. It catalyzes the formation of acetyl-CoA and oxaloacetate from citrate and CoA with concomitant hydrolysis of ATP to ADP and phosphate. Acetyl-CoA serves several critical biosynthetic pathways, including lipogenesis and cholesterogenesis. In nervous tissue, ATP citrate-lyase may be involved in the biosynthesis of acetylcholine.	Acute myeloid leukemia [40]	Overexpressed
			Prostate cancer [41]	Overexpressed
			Colon cancer [42]	Overexpressed
			Breast cancer [43]	Overexpressed
			Esophageal cancer [44]	Overexpressed
			Lung cancer [45]	Overexpressed
			Ovarian Cancer [46]	Overexpressed
			Endometrial cancer [47]	Overexpressed
			Thyroid cancer [48]	Overexpressed
			Hepatocellular carcinoma [49,50]	Overexpressed

Gene symbol	Official full name	Function	Relationship with any kind of cancer	Gene alteration and others
			Head and Neck Squamous Cell Carcinomas [51]	Radiosensitivity
JUP	Junction plakoglobin	This gene encodes a major cytoplasmic protein which is the only known constituent common to submembranous plaques of both desmosomes and intermediate junctions. This protein forms distinct complexes with cadherins and desmosomal cadherins and is a member of the catenin family since it contains a distinct repeating amino acid motif called the armadillo repeat [52]	Acute lymphoblastic leukemia [53]	Overexpressed
			Acute Mieloid Leukemia [54]	Overexpressed
			Ovarian cancer [55]	Overexpressed
			Gastric cancer [52]	Underexpressed
			Prostate cancer [56]	Overexpressed or underexpressed in different stages of disease
			Lung cancer [56,57]	Underexpressed
			Breast cancer [58,59]	Overexpressed
			Oral squamous cell carcinoma [60]	Overexpressed
			Colorectal cancer [61]	Overexpressed
KLHL10	Kelch like family member 10	The protein encoded by this gene belongs to the kelch repeat-containing family and contains an N-terminal BTB/POZ domain, a BACK domain, and six C-terminal kelch repeats. It may be a substrate-specific adapter of a CUL3-based E3 ubiquitin-protein ligase complex which mediates the ubiquitination and subsequent proteasomal degradation of target proteins during spermatogenesis.	None	None
KLHL11	Kelch like family member 11	KLHL11 translates the Component of a cullin-RING-based BCR (BTB-CUL3-RBX1)	None	None

Gene symbol	Official full name	Function	Relationship with any kind of cancer	Gene alteration and others
		E3 ubiquitin-protein ligase complex that mediates the ubiquitination of target proteins, leading most often to their proteasomal degradation.		
TTC25-ODAD4	Outer dynein arm docking complex subunit 4	This gene encodes a tetratricopeptide repeat domain-containing protein that localizes to ciliary axonmenes and plays a role in docking the outer dynein arm to cilia.	None	None
RTL10-C22orf29	Retrotransposon Gag like 10	The protein translates for *RTL10- C22orf29*, which could induce apoptosis in a BH3 domain-dependent manner. This gene is also involved in mitochondrial outer membrane permeabilization and regulation of mitochondrial membrane potential.	Acute chronic leukemia [62]	Overexpressed
TXNRD2	Thioredoxin reductase 2	The protein encoded by this gene belongs to the pyridine nucleotide-disulfide oxidoreductase family, and is a member of the thioredoxin (Trx) system. Three thioredoxin reductase (TrxR) isozymes are found in mammals. TrxRs are selenocysteine-containing flavoenzymes, which reduce thioredoxins, and other substrates, and play a key role in redox homeostasis.	Acute chronic leukemia [63]	Underexpressed
			Breast cancer [64]	Overexpressed
			Mieloma [65]	Overexpressed
COMT	Catechol-O-methyltransferase	*COMT* translates Catechol-O-methyltransferase that catalyzes the transfer of a methyl group from S-adenosylmethionine to catecholamines,	Acute lymphoblastic leukemia [66]	Variant rs4680 associated with patients who experienced hepatotoxicity

Gene symbol	Official full name	Function	Relationship with any kind of cancer	Gene alteration and others
		including the neurotransmitters dopamine, epinephrine, and norepinephrine. This O-methylation results in one of the major degradative pathways of the catecholamine transmitters.	Prostate cancer [67]	Underexpressed
ARVCF	ARVCF delta catenin family member	Armadillo Repeat gene deleted in Velo-Cardio-Facial syndrome (*ARVCF*) is a catenin family member. This family plays an essential role in forming adherens junction complexes, which are thought to facilitate communication between the inside and outside environments of the cell.	Non-small cell lung cancer [68]	Overexpressed
			Prostate Cancer [69]	Correlation of SNP "rs5993891" with prostate patients
			Ranal Cancer [70]	Overexpressed
GNB1L	G protein subunit beta 1 like	This gene encodes a G-protein beta-subunit-like polypeptide, a member of the WD repeat protein family. WD repeats are minimally conserved regions of approximately 40 amino acids typically bracketed by gly-his and trp-asp (GH-WD), which may facilitate the formation of heterotrimeric or multiprotein complexes. Members of this family are involved in a variety of cellular processes, including cell cycle progression, signal transduction, apoptosis, and gene regulation.	Hepatocellular Carcinomas [71]	High frequencies of somatic copy number variation (CNV)
TANGO2	Transport and golgi organization 2 homolog	This gene belongs to the transport and Golgi organization family, whose members are predicted to play roles in secretory protein loading in the endoplasmic reticulum.	Prostate cancer [72]	Overexpressed

1. Liang, C.; Zhao, Y.; Chen, C.; Huang, S.; Deng, T.; Zeng, X.; Tan, J.; Zha, X.; Chen, S.; Li, Y. Higher TOX Genes Expression Is Associated With Poor Overall Survival for Patients With Acute Myeloid Leukemia. *Front Oncol* **2021**, *11*, 740642, doi:10.3389/fonc.2021.740642.
2. Tessema, M.; Yingling, C.M.; Grimes, M.J.; Thomas, C.L.; Liu, Y.; Leng, S.; Joste, N.; Belinsky, S.A. Differential epigenetic regulation of TOX subfamily high mobility group box genes in lung and breast cancers. *PLoS One* **2012**, *7*, e34850, doi:10.1371/journal.pone.0034850.
3. Sun, T.; Liu, Q.; Wang, Y.; Deng, Y.; Zhang, D. MBD2 mediates renal cell apoptosis via activation of Tox4 during rhabdomyolysis-induced acute kidney injury. *J Cell Mol Med* **2021**, *25*, 4562-4571, doi:10.1111/jcmm.16207.
4. Vu, L.P.; Pickering, B.F.; Cheng, Y.; Zaccara, S.; Nguyen, D.; Minuesa, G.; Chou, T.; Chow, A.; Saletore, Y.; MacKay, M.; et al. The N(6)-methyladenosine (m(6)A)-forming enzyme METTL3 controls myeloid differentiation of normal hematopoietic and leukemia cells. *Nat Med* **2017**, *23*, 1369-1376, doi:10.1038/nm.4416.
5. Yankova, E.; Blackaby, W.; Albertella, M.; Rak, J.; De Braekeleer, E.; Tsagkogeorga, G.; Pilka, E.S.; Aspris, D.; Leggate, D.; Hendrick, A.G.; et al. Small-molecule inhibition of METTL3 as a strategy against myeloid leukaemia. *Nature* **2021**, *593*, 597-601, doi:10.1038/s41586-021-03536-w.
6. Wang, Q.; Guo, X.; Li, L.; Gao, Z.; Su, X.; Ji, M.; Liu, J. N(6)-methyladenosine METTL3 promotes cervical cancer tumorigenesis and Warburg effect through YTHDF1/HK2 modification. *Cell Death Dis* **2020**, *11*, 911, doi:10.1038/s41419-020-03071-y.
7. Chen, H.; Gao, S.; Liu, W.; Wong, C.C.; Wu, J.; Wu, J.; Liu, D.; Gou, H.; Kang, W.; Zhai, J.; et al. RNA N(6)-Methyladenosine Methyltransferase METTL3 Facilitates Colorectal Cancer by Activating the m(6)A-GLUT1-mTORC1 Axis and Is a Therapeutic Target. *Gastroenterology* **2021**, *160*, 1284-1300 e1216, doi:10.1053/j.gastro.2020.11.013.
8. Han, J.; Wang, J.Z.; Yang, X.; Yu, H.; Zhou, R.; Lu, H.C.; Yuan, W.B.; Lu, J.C.; Zhou, Z.J.; Lu, Q.; et al. METTL3 promote tumor proliferation of bladder cancer by accelerating pri-miR221/222 maturation in m6A-dependent manner. *Mol Cancer* **2019**, *18*, 110, doi:10.1186/s12943-019-1036-9.
9. Wang, Q.; Chen, C.; Ding, Q.; Zhao, Y.; Wang, Z.; Chen, J.; Jiang, Z.; Zhang, Y.; Xu, G.; Zhang, J.; et al. METTL3-mediated m(6)A modification of HDGF mRNA promotes gastric cancer progression and has prognostic significance. *Gut* **2020**, *69*, 1193-1205, doi:10.1136/gutjnl-2019-319639.
10. Chen, Y.; Pan, C.; Wang, X.; Xu, D.; Ma, Y.; Hu, J.; Chen, P.; Xiang, Z.; Rao, Q.; Han, X. Silencing of METTL3 effectively hinders invasion and metastasis of prostate cancer cells. *Theranostics* **2021**, *11*, 7640-7657, doi:10.7150/thno.61178.
11. Xia, T.; Wu, X.; Cao, M.; Zhang, P.; Shi, G.; Zhang, J.; Lu, Z.; Wu, P.; Cai, B.; Miao, Y.; et al. The RNA m6A methyltransferase METTL3 promotes pancreatic cancer cell proliferation and invasion. *Pathol Res Pract* **2019**, *215*, 152666, doi:10.1016/j.prp.2019.152666.
12. Bi, X.; Lv, X.; Liu, D.; Guo, H.; Yao, G.; Wang, L.; Liang, X.; Yang, Y. METTL3-mediated maturation of miR-126-5p promotes ovarian cancer progression via PTEN-mediated PI3K/Akt/mTOR pathway. *Cancer Gene Ther* **2021**, *28*, 335-349, doi:10.1038/s41417-020-00222-3.
13. Han, H.; Yang, C.; Zhang, S.; Cheng, M.; Guo, S.; Zhu, Y.; Ma, J.; Liang, Y.; Wang, L.; Zheng, S.; et al. METTL3-mediated m(6)A mRNA modification promotes esophageal cancer initiation and progression via Notch signaling pathway. *Mol Ther Nucleic Acids* **2021**, *26*, 333-346, doi:10.1016/j.omtn.2021.07.007.

14. Wang, Q.; Geng, W.; Guo, H.; Wang, Z.; Xu, K.; Chen, C.; Wang, S. Emerging role of RNA methyltransferase METTL3 in gastrointestinal cancer. *J Hematol Oncol* **2020**, *13*, 57, doi:10.1186/s13045-020-00895-1.
15. He, J.; Zhou, M.; Yin, J.; Wan, J.; Chu, J.; Jia, J.; Sheng, J.; Wang, C.; Yin, H.; He, F. METTL3 restrains papillary thyroid cancer progression via m(6)A/c-Rel/IL-8-mediated neutrophil infiltration. *Mol Ther* **2021**, *29*, 1821-1837, doi:10.1016/j.ymthe.2021.01.019.
16. Hu, Y.; Li, Y.; Huang, Y.; Jin, Z.; Wang, C.; Wang, H.; Xu, J. METTL3 regulates the malignancy of cervical cancer via post-transcriptional regulation of RAB2B. *Eur J Pharmacol* **2020**, *879*, 173134, doi:10.1016/j.ejphar.2020.173134.
17. Jin, J.; Wu, Y.; Zhou, D.; Sun, Q.; Wang, W. miR448 targets Rab2B and is pivotal in the suppression of pancreatic cancer. *Oncol Rep* **2018**, *40*, 1379-1389, doi:10.3892/or.2018.6562.
18. Chen, B.; Jiang, L.; Zhong, M.L.; Li, J.F.; Li, B.S.; Peng, L.J.; Dai, Y.T.; Cui, B.W.; Yan, T.Q.; Zhang, W.N.; et al. Identification of fusion genes and characterization of transcriptome features in T-cell acute lymphoblastic leukemia. *Proc Natl Acad Sci U S A* **2018**, *115*, 373-378, doi:10.1073/pnas.1717125115.
19. Chai, L. The role of HSAL (SALL) genes in proliferation and differentiation in normal hematopoiesis and leukemogenesis. *Transfusion* **2011**, *51 Suppl 4*, 87S-93S, doi:10.1111/j.1537-2995.2011.03371.x.
20. Luo, J.; Wang, W.; Tang, Y.; Zhou, D.; Gao, Y.; Zhang, Q.; Zhou, X.; Zhu, H.; Xing, L.; Yu, J. mRNA and methylation profiling of radioresistant esophageal cancer cells: the involvement of Sall2 in acquired aggressive phenotypes. *J Cancer* **2017**, *8*, 646-656, doi:10.7150/jca.15652.
21. Ye, L.; Lin, C.; Wang, X.; Li, Q.; Li, Y.; Wang, M.; Zhao, Z.; Wu, X.; Shi, D.; Xiao, Y.; et al. Epigenetic silencing of SALL2 confers tamoxifen resistance in breast cancer. *EMBO Mol Med* **2019**, *11*, e10638, doi:10.15252/emmm.201910638.
22. Alagaratnam, S.; Lind, G.E.; Kraggerud, S.M.; Lothe, R.A.; Skotheim, R.I. The testicular germ cell tumour transcriptome. *Int J Androl* **2011**, *34*, e133-150; discussion e150-131, doi:10.1111/j.1365-2605.2011.01169.x.
23. Imai, A.; Mochizuki, D.; Misawa, Y.; Nakagawa, T.; Endo, S.; Mima, M.; Yamada, S.; Kawasaki, H.; Kanazawa, T.; Misawa, K. SALL2 Is a Novel Prognostic Methylation Marker in Patients with Oral Squamous Carcinomas: Associations with SALL1 and SALL3 Methylation Status. *DNA Cell Biol* **2019**, *38*, 678-687, doi:10.1089/dna.2018.4597.
24. Suva, M.L.; Rheinbay, E.; Gillespie, S.M.; Patel, A.P.; Wakimoto, H.; Rabkin, S.D.; Riggi, N.; Chi, A.S.; Cahill, D.P.; Nahed, B.V.; et al. Reconstructing and reprogramming the tumor-propagating potential of glioblastoma stem-like cells. *Cell* **2014**, *157*, 580-594, doi:10.1016/j.cell.2014.02.030.
25. Sung, C.K.; Li, D.; Andrews, E.; Drapkin, R.; Benjamin, T. Promoter methylation of the SALL2 tumor suppressor gene in ovarian cancers. *Mol Oncol* **2013**, *7*, 419-427, doi:10.1016/j.molonc.2012.11.005.
26. He, X.Y.; Yang, W.M.; Tang, W.T.; Ma, R.; Sun, Y.P.; Wang, P.; Yao, X.S. TRAV gene expression in PBMCs and TILs in patients with breast cancer analyzed by a DNA melting curve (FQ-PCR) technique for TCR alpha chain CDR3 spectratyping. *Neoplasma* **2012**, *59*, 693-699, doi:10.4149/neo_2012_088.

27. Sun, Z.; Dong, J.; Zhang, S.; Hu, Z.; Cheng, K.; Li, K.; Xu, B.; Ye, M.; Nie, Y.; Fan, D.; et al. Identification of chemoresistance-related cell-surface glycoproteins in leukemia cells and functional validation of candidate glycoproteins. *J Proteome Res* **2014**, *13*, 1593-1601, doi:10.1021/pr4010822.
28. Wang, R.G.; Zhang, D.; Zhao, C.H.; Wang, Q.L.; Qu, H.; He, Q.S. FKBP10 functioned as a cancer-promoting factor mediates cell proliferation, invasion, and migration via regulating PI3K signaling pathway in stomach adenocarcinoma. *Kaohsiung J Med Sci* **2020**, *36*, 311-317, doi:10.1002/kjm2.12174.
29. Gong, L.B.; Zhang, C.; Yu, R.X.; Li, C.; Fan, Y.B.; Liu, Y.P.; Qu, X.J. FKBP10 Acts as a New Biomarker for Prognosis and Lymph Node Metastasis of Gastric Cancer by Bioinformatics Analysis and in Vitro Experiments. *Onco Targets Ther* **2020**, *13*, 7399-7409, doi:10.2147/OTT.S253154.
30. Ramadori, G.; Ioris, R.M.; Villanyi, Z.; Firnkes, R.; Panasenko, O.O.; Allen, G.; Konstantinidou, G.; Aras, E.; Brenachot, X.; Biscotti, T.; et al. FKBP10 Regulates Protein Translation to Sustain Lung Cancer Growth. *Cell Rep* **2020**, *30*, 3851-3863 e3856, doi:10.1016/j.celrep.2020.02.082.
31. Sun, Z.; Qin, X.; Fang, J.; Tang, Y.; Fan, Y. Multi-Omics Analysis of the Expression and Prognosis for FKBP Gene Family in Renal Cancer. *Front Oncol* **2021**, *11*, 697534, doi:10.3389/fonc.2021.697534.
32. Cai, H.Q.; Zhang, M.J.; Cheng, Z.J.; Yu, J.; Yuan, Q.; Zhang, J.; Cai, Y.; Yang, L.Y.; Zhang, Y.; Hao, J.J.; et al. FKBP10 promotes proliferation of glioma cells via activating AKT-CREB-PCNA axis. *J Biomed Sci* **2021**, *28*, 13, doi:10.1186/s12929-020-00705-3.
33. Quinn, M.C.; Wojnarowicz, P.M.; Pickett, A.; Provencher, D.M.; Mes-Masson, A.M.; Davis, E.C.; Tonin, P.N. FKBP10/FKBP65 expression in high-grade ovarian serous carcinoma and its association with patient outcome. *Int J Oncol* **2013**, *42*, 912-920, doi:10.3892/ijo.2013.1797.
34. Wang, L.; Zeng, D.; Wang, Q.; Liu, L.; Lu, T.; Gao, Y. Screening and Identification of Novel Potential Biomarkers for Breast Cancer Brain Metastases. *Front Oncol* **2021**, *11*, 784096, doi:10.3389/fonc.2021.784096.
35. Hao, L.; Pang, K.; Pang, H.; Zhang, J.; Zhang, Z.; He, H.; Zhou, R.; Shi, Z.; Han, C. Knockdown of P3H4 inhibits proliferation and invasion of bladder cancer. *Aging (Albany NY)* **2020**, *12*, 2156-2168, doi:10.18632/aging.102732.
36. Jin, X.; Zhou, H.; Song, J.; Cui, H.; Luo, Y.; Jiang, H. P3H4 Overexpression Serves as a Prognostic Factor in Lung Adenocarcinoma. *Comput Math Methods Med* **2021**, *2021*, 9971353, doi:10.1155/2021/9971353.
37. Wan, B.; Zeng, Q.; Tang, X.Z.; Tang, Y.X. P3H4 affects renal carcinoma through up-regulating miR-1/133a. *Eur Rev Med Pharmacol Sci* **2018**, *22*, 5180-5186, doi:10.26355/eurrev_201808_15714.
38. Li, C.; Wang, J.; Hao, J.; Dong, B.; Li, Y.; Zhu, X.; Ding, J.; Ren, S.; Zhao, H.; Wu, S.; et al. Reduced cytosolic carboxypeptidase 6 (CCP6) level leads to accumulation of serum polyglutamylated DNAJC7 protein: A potential biomarker for renal cell carcinoma early detection. *Oncotarget* **2016**, *7*, 22385-22396, doi:10.18632/oncotarget.8107.
39. Zorniak, M.; Clark, P.A.; Leeper, H.E.; Tipping, M.D.; Francis, D.M.; Kozak, K.R.; Salamat, M.S.; Kuo, J.S. Differential expression of 2',3'-cyclic-nucleotide 3'-phosphodiesterase and neural lineage markers correlate with glioblastoma xenograft infiltration and patient survival. *Clin Cancer Res* **2012**, *18*, 3628-3636, doi:10.1158/1078-0432.CCR-12-0339.

40. Wang, J.; Ye, W.; Yan, X.; Guo, Q.; Ma, Q.; Lin, F.; Huang, J.; Jin, J. Low expression of ACLY associates with favorable prognosis in acute myeloid leukemia. *J Transl Med* **2019**, *17*, 149, doi:10.1186/s12967-019-1884-5.
41. Shah, S.; Carriveau, W.J.; Li, J.; Campbell, S.L.; Kopinski, P.K.; Lim, H.W.; Daurio, N.; Trefely, S.; Won, K.J.; Wallace, D.C.; et al. Targeting ACLY sensitizes castration-resistant prostate cancer cells to AR antagonism by impinging on an ACLY-AMPK-AR feedback mechanism. *Oncotarget* **2016**, *7*, 43713-43730, doi:10.18632/oncotarget.9666.
42. Wen, J.; Min, X.; Shen, M.; Hua, Q.; Han, Y.; Zhao, L.; Liu, L.; Huang, G.; Liu, J.; Zhao, X. ACLY facilitates colon cancer cell metastasis by CTNNB1. *J Exp Clin Cancer Res* **2019**, *38*, 401, doi:10.1186/s13046-019-1391-9.
43. Chen, Y.; Li, K.; Gong, D.; Zhang, J.; Li, Q.; Zhao, G.; Lin, P. ACLY: A biomarker of recurrence in breast cancer. *Pathol Res Pract* **2020**, *216*, 153076, doi:10.1016/j.prp.2020.153076.
44. Guo, H.; Wang, B.; Xu, K.; Nie, L.; Fu, Y.; Wang, Z.; Wang, Q.; Wang, S.; Zou, X. m(6)A Reader HNRNPA2B1 Promotes Esophageal Cancer Progression via Up-Regulation of ACLY and ACC1. *Front Oncol* **2020**, *10*, 553045, doi:10.3389/fonc.2020.553045.
45. Merino Salvador, M.; Gomez de Cedron, M.; Moreno Rubio, J.; Falagan Martinez, S.; Sanchez Martinez, R.; Casado, E.; Ramirez de Molina, A.; Sereno, M. Lipid metabolism and lung cancer. *Crit Rev Oncol Hematol* **2017**, *112*, 31-40, doi:10.1016/j.critrevonc.2017.02.001.
46. Wei, X.; Shi, J.; Lin, Q.; Ma, X.; Pang, Y.; Mao, H.; Li, R.; Lu, W.; Wang, Y.; Liu, P. Targeting ACLY Attenuates Tumor Growth and Acquired Cisplatin Resistance in Ovarian Cancer by Inhibiting the PI3K-AKT Pathway and Activating the AMPK-ROS Pathway. *Front Oncol* **2021**, *11*, 642229, doi:10.3389/fonc.2021.642229.
47. Dai, M.; Yang, B.; Chen, J.; Liu, F.; Zhou, Y.; Zhou, Y.; Xu, Q.; Jiang, S.; Zhao, S.; Li, X.; et al. Nuclear-translocation of ACLY induced by obesity-related factors enhances pyrimidine metabolism through regulating histone acetylation in endometrial cancer. *Cancer Lett* **2021**, *513*, 36-49, doi:10.1016/j.canlet.2021.04.024.
48. Huang, S.S.; Tsai, C.H.; Kuo, C.Y.; Li, Y.S.; Cheng, S.P. ACLY inhibitors induce apoptosis and potentiate cytotoxic effects of sorafenib in thyroid cancer cells. *Endocrine* **2022**, *78*, 85-94, doi:10.1007/s12020-022-03124-6.
49. Liu, D.; Zhang, T.; Chen, X.; Zhang, B.; Wang, Y.; Xie, M.; Ji, X.; Sun, M.; Huang, W.; Xia, L. Correction to: ONECUT2 facilitates hepatocellular carcinoma metastasis by transcriptionally upregulating FGF2 and ACLY. *Cell Death Dis* **2021**, *13*, 28, doi:10.1038/s41419-021-04475-0.
50. Xu, Y.; Zhang, Z.; Xu, D.; Yang, X.; Zhou, L.; Zhu, Y. Identification and integrative analysis of ACLY and related gene panels associated with immune microenvironment reveal prognostic significance in hepatocellular carcinoma. *Cancer Cell Int* **2021**, *21*, 409, doi:10.1186/s12935-021-02108-2.
51. Gottgens, E.L.; van den Heuvel, C.N.; de Jong, M.C.; Kaanders, J.H.; Leenders, W.P.; Ansems, M.; Bussink, J.; Span, P.N. ACLY (ATP Citrate Lyase) Mediates Radioresistance in Head and Neck Squamous Cell Carcinomas and is a Novel Predictive Radiotherapy Biomarker. *Cancers (Basel)* **2019**, *11*, doi:10.3390/cancers11121971.
52. Chen, Y.; Yang, L.; Qin, Y.; Liu, S.; Qiao, Y.; Wan, X.; Zeng, H.; Tang, X.; Liu, M.; Hou, Y. Effects of differential distributed-JUP on the malignancy of gastric cancer. *J Adv Res* **2021**, *28*, 195-208, doi:10.1016/j.jare.2020.06.026.

53. Luong-Gardiol, N.; Siddiqui, I.; Pizzitola, I.; Jeevan-Raj, B.; Charmoy, M.; Huang, Y.; Irmisch, A.; Curtet, S.; Angelov, G.S.; Danilo, M.; et al. gamma-Catenin-Dependent Signals Maintain BCR-ABL1(+) B Cell Acute Lymphoblastic Leukemia. *Cancer Cell* **2019**, *35*, 649-663 e610, doi:10.1016/j.ccell.2019.03.005.
54. Qian, J.; Huang, X.; Zhang, Y.; Ye, X.; Qian, W. gamma-Catenin Overexpression in AML Patients May Promote Tumor Cell Survival via Activation of the Wnt/beta-Catenin Axis. *Onco Targets Ther* **2020**, *13*, 1265-1276, doi:10.2147/OTT.S230873.
55. Weiland, F.; Lokman, N.A.; Klingler-Hoffmann, M.; Jobling, T.; Stephens, A.N.; Sundfeldt, K.; Hoffmann, P.; Oehler, M.K. Ovarian Blood Sampling Identifies Junction Plakoglobin as a Novel Biomarker of Early Ovarian Cancer. *Front Oncol* **2020**, *10*, 1767, doi:10.3389/fonc.2020.01767.
56. Spethmann, T.; Bockelmann, L.C.; Labitzky, V.; Ahlers, A.K.; Schroder-Schwarz, J.; Bonk, S.; Simon, R.; Sauter, G.; Huland, H.; Kypta, R.; et al. Opposing prognostic relevance of junction plakoglobin in distinct prostate cancer patient subsets. *Mol Oncol* **2021**, *15*, 1956-1969, doi:10.1002/1878-0261.12922.
57. Sang, Y.; Sun, L.; Wu, Y.; Yuan, W.; Liu, Y.; Li, S.W. Histone deacetylase 7 inhibits plakoglobin expression to promote lung cancer cell growth and metastasis. *Int J Oncol* **2019**, *54*, 1112-1122, doi:10.3892/ijo.2019.4682.
58. Lu, L.; Zeng, H.; Gu, X.; Ma, W. Circulating tumor cell clusters-associated gene plakoglobin and breast cancer survival. *Breast Cancer Res Treat* **2015**, *151*, 491-500, doi:10.1007/s10549-015-3416-1.
59. Xie, N.; Hu, Z.; Tian, C.; Xiao, H.; Liu, L.; Yang, X.; Li, J.; Wu, H.; Lu, J.; Gao, J.; et al. In Vivo Detection of CTC and CTC Plakoglobin Status Helps Predict Prognosis in Patients with Metastatic Breast Cancer. *Pathol Oncol Res* **2020**, *26*, 2435-2442, doi:10.1007/s12253-020-00847-7.
60. Fang, J.; Xiao, L.; Zhang, Q.; Peng, Y.; Wang, Z.; Liu, Y. Junction plakoglobin, a potential prognostic marker of oral squamous cell carcinoma, promotes proliferation, migration and invasion. *J Oral Pathol Med* **2020**, *49*, 30-38, doi:10.1111/jop.12952.
61. Nagel, J.M.; Lahm, H.; Ofner, A.; Goke, B.; Kolligs, F.T. gamma-Catenin acts as a tumor suppressor through context-dependent mechanisms in colorectal cancer. *Int J Colorectal Dis* **2017**, *32*, 1243-1251, doi:10.1007/s00384-017-2846-0.
62. Zhu, H.J.; Liu, L.; Fan, L.; Zhang, L.N.; Fang, C.; Zou, Z.J.; Li, J.Y.; Xu, W. The BH3-only protein Puma plays an essential role in p53-mediated apoptosis of chronic lymphocytic leukemia cells. *Leuk Lymphoma* **2013**, *54*, 2712-2719, doi:10.3109/10428194.2013.787613.
63. Barrow, T.M.; Wong Doo, N.; Milne, R.L.; Giles, G.G.; Willmore, E.; Strathdee, G.; Byun, H.M. Analysis of retrotransposon subfamily DNA methylation reveals novel early epigenetic changes in chronic lymphocytic leukemia. *Haematologica* **2021**, *106*, 98-110, doi:10.3324/haematol.2019.228478.
64. Yang, D.; Guo, Q.; Liang, Y.; Zhao, Y.; Tian, X.; Ye, Y.; Tian, J.; Wu, T.; Lu, N. Wogonin induces cellular senescence in breast cancer via suppressing TXNRD2 expression. *Arch Toxicol* **2020**, *94*, 3433-3447, doi:10.1007/s00204-020-02842-y.
65. Fink, E.E.; Mannava, S.; Bagati, A.; Bianchi-Smiraglia, A.; Nair, J.R.; Moparthy, K.; Lipchick, B.C.; Drokov, M.; Utley, A.; Ross, J.; et al. Mitochondrial thioredoxin reductase regulates major cytotoxicity pathways of proteasome inhibitors in multiple myeloma cells. *Leukemia* **2016**, *30*, 104-111, doi:10.1038/leu.2015.190.

66. Cao, M.; Yin, D.; Qin, Y.; Liao, F.; Su, Y.; Xia, X.; Gao, J.; Zhu, Y.; Zhang, W.; Shu, Y.; et al. Screening of Novel Pharmacogenetic Candidates for Mercaptopurine-Induced Toxicity in Patients With Acute Lymphoblastic Leukemia. *Front Pharmacol* **2020**, *11*, 267, doi:10.3389/fphar.2020.00267.
67. Hashimoto, Y.; Shiina, M.; Maekawa, S.; Kato, T.; Shahryari, V.; Kulkarni, P.; Dasgupta, P.; Yamamura, S.; Saini, S.; Tabatabai, Z.L.; et al. Suppressor effect of catechol-O-methyltransferase gene in prostate cancer. *PLoS One* **2021**, *16*, e0253877, doi:10.1371/journal.pone.0253877.
68. Zhang, D.; Tang, N.; Liu, Y.; Wang, E.H. ARVCF expression is significantly correlated with the malignant phenotype of non-small cell lung cancer. *Mol Carcinog* **2015**, *54 Suppl 1*, E185-191, doi:10.1002/mc.22281.
69. Lin, D.W.; FitzGerald, L.M.; Fu, R.; Kwon, E.M.; Zheng, S.L.; Kolb, S.; Wiklund, F.; Stattin, P.; Isaacs, W.B.; Xu, J.; et al. Genetic variants in the LEPR, CRY1, RNASEL, IL4, and ARVCF genes are prognostic markers of prostate cancer-specific mortality. *Cancer Epidemiol Biomarkers Prev* **2011**, *20*, 1928-1936, doi:10.1158/1055-9965.EPI-11-0236.
70. Walter, B.; Berger, I.; Hofmann, I. The proteins ARVCF and p0071 in renal cell carcinomas and their potential use in the diagnosis of renal tumours. *Histopathology* **2009**, *55*, 761-764, doi:10.1111/j.1365-2559.2009.03444.x.
71. Nalesnik, M.A.; Tseng, G.; Ding, Y.; Xiang, G.S.; Zheng, Z.L.; Yu, Y.; Marsh, J.W.; Michalopoulos, G.K.; Luo, J.H. Gene deletions and amplifications in human hepatocellular carcinomas: correlation with hepatocyte growth regulation. *Am J Pathol* **2012**, *180*, 1495-1508, doi:10.1016/j.ajpath.2011.12.021.
72. Karyadi, D.M.; Geybels, M.S.; Karlins, E.; Decker, B.; McIntosh, L.; Hutchinson, A.; Kolb, S.; McDonnell, S.K.; Hicks, B.; Middha, S.; et al. Whole exome sequencing in 75 high-risk families with validation and replication in independent case-control studies identifies TANGO2, OR5H14, and CHAD as new prostate cancer susceptibility genes. *Oncotarget* **2017**, *8*, 1495-1507, doi:10.18632/oncotarget.13646.
