# Supplementary material for: Comparative Genomic Hybridization and Transcriptome Sequencing Reveal Genes with Gain in Acute Lymphoblastic Leukemia: JUP Expression Emerges as a Survival-Related Gene
Source: Diagnostics (Basel). 2022 Nov 14;12(11):2788. doi: 10.3390/diagnostics12112788 (PMC9689318; doi:10.3390/diagnostics12112788)
Supplement: Supplementary file 1 [file diagnostics-12-02788-s001.zip › Supplementary Table S2.pdf]

**Supplementary Table S2. Data of DNA copy number normalized to Log2 of chromosomes 14, 17, and 22.** The values in yellow represent zones of the chromosome where we found a gain in T-ALL and B-ALL samples. Chr: chromosome, Start: starting position, End: finishing position, GSM: sample identifier.

| T-ALL Samples | | | | | | | | | | | |
|---|---|---|---|---|---|---|---|---|---|---|---|
| **Chr** | **Start** | **End** | **GSM1963457** | **GSM1963456** | **GSM1963455** | **GSM1963454** | **GSM1963453** | **GSM1963452** | **GSM1963451** | **GSM1963450** | **GSM1963449** |
| chr14 | 21444377 | 21444436 | 1.81E-01 | -2.79E-02 | 5.12E-01 | -1.35E-01 | 2.27E-01 | 1.52E-01 | -5.04E-01 | 2.85E-01 | 2.99E-01 |
| chr14 | 21646729 | 21646788 | 1.87E-01 | 4.59E-01 | -1.75E+00 | -3.99E-01 | -4.43E-01 | -2.28E-01 | 7.91E-02 | 4.11E-01 | 1.02E-01 |
| **B-ALL Samples** | | | | | | | | | | | |
| **Chr** | **Start** | **End** | **GSM1963407** | **GSM1963406** | **GSM1963405** | **GSM1963404** | **GSM1963403** | **GSM1963402** | **GSM1963401** | **GSM1963400** | **GSM1963399** |
| chr14 | 21444377 | 21444436 | -4.46E-01 | 1.89E-01 | 7.96E-02 | 2.49E-01 | 4.30E-01 | -1.22E-01 | -4.25E-02 | -4.44E-01 | 4.81E-01 |
| chr14 | 21646729 | 21646788 | -2.74E-01 | 1.84E-01 | 7.18E-02 | 3.05E-01 | 5.66E-01 | 9.03E-02 | 3.54E-03 | -3.85E-01 | 2.78E-01 |
| **T-ALL Samples** | | | | | | | | | | | |
| **Chr** | **Start** | **End** | **GSM1963457** | **GSM1963456** | **GSM1963455** | **GSM1963454** | **GSM1963453** | **GSM1963452** | **GSM1963451** | **GSM1963450** | **GSM1963449** |
| chr17 | 41755952 | 41756011 | -4.75E-01 | 3.78E-02 | 3.73E-01 | 3.19E-01 | -1.43E-02 | 4.11E-01 | 6.96E-01 | 1.57E-01 | 1.56E-01 |
| chr17 | 41788252 | 41788311 | 6.75E-02 | -1.44E-01 | 7.47E-01 | 3.61E-01 | 1.76E-02 | -1.60E-01 | 2.81E-01 | -2.25E-01 | 5.06E-01 |
| chr17 | 41819694 | 41819753 | -4.96E-01 | -1.87E-01 | 7.00E-01 | 3.95E-01 | -1.26E-01 | 5.49E-02 | 1.95E-01 | -3.12E-01 | 2.57E-01 |
| chr17 | 41854798 | 41854857 | -5.44E-01 | -6.43E-03 | 5.36E-01 | 5.25E-01 | 3.81E-01 | 1.64E-01 | 1.58E-01 | -5.98E-01 | -2.68E-01 |
| chr17 | 41876006 | 41876065 | -4.54E-01 | -3.51E-01 | 4.91E-01 | 3.46E-01 | 2.72E-01 | 7.99E-02 | 8.81E-02 | -6.14E-01 | -4.35E-02 |
| chr17 | 41958730 | 41958789 | -5.01E-01 | -2.22E-02 | 7.21E-02 | 2.60E-01 | 1.01E-01 | 4.05E-01 | 6.29E-01 | -5.55E-03 | 3.65E-01 |
| chr17 | 41999354 | 41999413 | -3.01E-01 | -2.59E-01 | 5.92E-01 | 4.25E-01 | 7.19E-02 | 2.35E-01 | 6.03E-01 | 9.03E-02 | 1.13E-01 |
| chr17 | 42005133 | 42005192 | -4.48E-01 | 9.57E-02 | 5.07E-01 | 6.10E-01 | 1.72E-01 | -1.97E-01 | 2.21E-01 | -3.51E-01 | 1.38E-01 |
| **B-ALL Samples** | | | | | | | | | | | |
| **Chr** | **Start** | **End** | **GSM1963407** | **GSM1963406** | **GSM1963405** | **GSM1963404** | **GSM1963403** | **GSM1963402** | **GSM1963401** | **GSM1963400** | **GSM1963399** |
| chr17 | 41755952 | 41756011 | 1.55E-01 | -8.37E-02 | -4.26E-01 | -3.83E-01 | -2.01E-01 | 1.59E-04 | -6.93E-01 | -4.73E-01 | 6.89E-01 |
| chr17 | 41788252 | 41788311 | -1.03E-02 | 6.16E-01 | -1.17E-02 | -2.09E-01 | -1.83E-01 | 2.79E-01 | -2.55E-01 | -7.88E-04 | 4.83E-01 |
| chr17 | 41819694 | 41819753 | -3.70E-01 | 2.68E-01 | -2.53E-01 | 7.31E-02 | -2.95E-01 | -5.47E-01 | -6.98E-01 | -6.97E-01 | 9.96E-01 |
| chr17 | 41854798 | 41854857 | -5.97E-01 | 6.92E-02 | -5.82E-01 | -4.24E-01 | -5.48E-01 | -6.81E-01 | -8.59E-01 | -6.52E-01 | 1.14E+00 |
| chr17 | 41876006 | 41876065 | -7.22E-01 | 3.88E-01 | -6.13E-01 | 5.58E-03 | -2.76E-01 | -7.16E-01 | -6.07E-01 | -3.28E-01 | 1.17E+00 |
| chr17 | 41958730 | 41958789 | 8.66E-02 | -1.38E-02 | -1.79E-01 | -4.45E-01 | -5.39E-01 | -1.57E-01 | -5.68E-01 | -6.78E-01 | 4.61E-01 |

| | | | | | | | | | | | |
|---|---|---|---|---|---|---|---|---|---|---|---|
| chr17 | 41999354 | 41999413 | 3.17E-01 | 2.21E-01 | -4.66E-01 | -4.39E-01 | -4.95E-01 | -4.38E-01 | -6.51E-01 | -2.05E-02 | 7.85E-01 |
| chr17 | 42005133 | 42005192 | -2.77E-02 | 4.13E-01 | -4.67E-01 | -2.91E-01 | -6.24E-01 | -3.66E-01 | -3.44E-01 | -5.14E-01 | 5.91E-01 |
| **T-ALL Samples** | | | | | | | | | | | |
| **Chr** | **Start** | **End** | **GSM1963457** | **GSM1963456** | **GSM1963455** | **GSM1963454** | **GSM1963453** | **GSM1963452** | **GSM1963451** | **GSM1963450** | **GSM1963449** |
| chr22 | 20026630 | 20026689 | -2.16E-01 | -8.50E-01 | -1.96E+00 | -9.70E-01 | 1.14E-01 | 1.09E-01 | 1.22E-01 | -5.38E-01 | 2.08E-01 |
| chr22 | 20037371 | 20037430 | -7.03E-01 | -3.99E-01 | -8.94E-01 | -3.78E-01 | -7.05E-02 | 8.93E-01 | 3.28E-01 | -7.28E-01 | 1.65E-01 |
| **B-ALL Samples** | | | | | | | | | | | |
| **Chr** | **Start** | **End** | **GSM1963407** | **GSM1963406** | **GSM1963405** | **GSM1963404** | **GSM1963403** | **GSM1963402** | **GSM1963401** | **GSM1963400** | **GSM1963399** |
| chr22 | 20026630 | 20026689 | -1.02E-01 | -3.15E-01 | -4.36E-01 | 2.37E-01 | 4.23E-01 | 5.49E-01 | 8.45E-02 | -8.78E-02 | -5.45E-01 |
| chr22 | 20037371 | 20037430 | 1.87E-01 | -1.16E-01 | -7.11E-01 | -7.12E-02 | 1.02E+00 | 3.05E-01 | -4.44E-01 | -6.37E-01 | -2.53E-01 |
